# Neurobiological insights into the effects of ultra-processed food on lipid metabolism and associated mental health conditions: a scoping review

**DOI:** 10.3389/fnut.2025.1754492

**Published:** 2026-01-21

**Authors:** Emily Poon, Christine Li, Daniel Schweitzer, Isaac Akefe

**Affiliations:** 1 Medical School, Faculty of Medicine, University of Queensland, Brisbane, QLD, Australia; 2 Centre for Neurosciences, Mater Hospital, South Brisbane, QLD, Australia; 3 CDU-Menzies School of Medicine, Charles Darwin University, Darwin, NT, Australia

**Keywords:** anxiety, autism, depression, eating disorders, food addiction, lipid metabolism, mental health, neuroinflammation

## Abstract

**Background:**

Ultra-processed foods (UPFs) account for approximately 38% of the adult diet, corresponding with a global increase in the prevalence of mental illnesses. Understanding the relationship between UPF consumption and mental health is crucial for public health and clinical practice.

**Objectives:**

To uncover the association between consumption of ultra-processed food (UPF), dysregulated lipid metabolism, and increased risk of mental illnesses, including depression, anxiety, attention-deficit/hyperactivity disorder (ADHD), autism spectrum disorder (ASD), eating disorders (ED), and food addiction (FA). In addition, this review explores the potential biological and behavioral mechanisms that may underlie these associations for each disorder.

**Methods:**

Following the PRISMA extension for scoping reviews guideline, a comprehensive search was conducted across PubMed, Web of Science, and EMBASE databases. The retrieved records, screened using Covidence, included English-language studies published between 2020 and 2025 that involved participants without significant comorbidities. Relevant data on associations and proposed mechanisms were extracted and synthesized using a narrative approach.

**Results:**

UPF consumption was associated with dysregulated lipid metabolism and increased risk of Anxiety, Depression, ADHD, Autism, ED, and FA. Dose-dependent increases in risk were identified in all mental illnesses except for autism. Proposed mechanisms for all these increased risks included systemic low-grade inflammation, alterations in neuronal signaling, particularly dopamine and serotonin signaling pathways, and the influence of UPF additives on neurochemical regulation.

**Conclusion:**

There is a strong association between UPF consumption, disrupted lipid metabolism and increased risk of mental disorder in populations without significant comorbidities. Diets rich in minimally processed foods appear protective. The findings support the potential of public health initiatives aimed at reducing UPF consumption to mitigate the mental health burden. Future studies should focus on mechanistic pathways, UPF and minimally processed food consumption patterns to provide evidence for targeted dietary and policy interventions that improve health outcomes.

## 1 Introduction

The prevalence of ultra-processed foods (UPFs) is rapidly increasing across both developed and developing nations, which may in part be due to their availability, low price and additives which make them highly palatable (1). The NOVA classification system categorizes foods according to their level of processing. Based on this classification scheme, UPFs are defined as foods or beverages that are industrially formulated from food constituents and additives that rarely contain any whole foods (2). Common UPFs include deep-fried foods, packaged snacks, soft drinks, and instant meals (3). These foods are generally nutrient-poor but contain higher amounts of fat, sugar and additives, including preservatives, artificial flavors [such as monosodium glutamate (MSG)] and dyes (2, 4). In Australia, UPFs accounted for, on average, 38.8% of the adult diet (5). Based on findings from recent studies, the consumption of UPF has been increasingly implicated across a myriad of non-communicable diseases, such as cardiovascular disease, neurological disorders, type 2 diabetes, and cancers (6–8).

Although the prevalence of mental illness has increased over recent years, the reasons for the increased prevalence of mental disorders remain unclear. It has been well-established that most mental disorders are caused by an interplay of a range of factors, including genetic, early developmental factors, environmental factors, and, in some cases, an initiating or precipitating factor (9, 10). The increased prevalence of mental disorders may also be attributable to a range of factors, including increased awareness about mental disorders, reduced levels of stigma, as well as alternative methods of measurement and recording of mental disorders, which have led to an increased level of reporting of mental disorders (11).

The global burden of mental disorders is significant, with more than one billion individuals estimated to be living with a mental disorder as of 2021 (12). The most common mental disorders are anxiety and depressive disorders, which together account for over two-thirds of all mental health conditions (12). The prevalence of mental disorders continues to increase, potentially driven by a range of societal and social factors, such as the COVID-19 pandemic (13).

Mental illness in Australia is associated with significant morbidity. It accounts for approximately 15% of the total disability-adjusted life years (DALYs). It is the second most significant contributor to overall disability, only behind cancer (14). Over the 2022–2023 period, a total of $13.2 billion was collectively spent on mental health-related services by the government and private health services in Australia, with an estimated total cost of $220 billion when accounting for lost productivity (15). At the individual level, several previous studies have shown that mental disorders have the potential to contribute to a range of other problems, including an increased suicide risk as well as reduced quality of life across multiple domains, including impairments in physical and social functioning (16). Hence, it is important to reduce the prevalence of mental illness and understand the different risk factors associated with the development of mental disorders in view of their significant impacts, both at an individual and at a community level (16, 17). Creating a more nuanced understanding of its impact, at a public health and economic level, will be important for developing public policy around cost-effective and evidence-based.

Previous research has established an association between specific dietary patterns and the risk of mental disorders, with multiple studies demonstrating that individual dietary components, including the type and intake levels of saturated fats and sugars, contribute to the development of mental health disorders (18, 19). With the increasing popularity and consumption of UPFs worldwide, the role of food processing is emerging as a central area of investigation and discussion across various studies (1, 2). To date, there have only been a few studies that have established a clear association and linkage between the level of UPF consumption and the development of mental illness, particularly in terms of the development of mood and anxiety disorders, such as major depressive disorder (MDD) (20, 21). In fact, frequent UPF consumption was found to be associated with an increased cross-sectional risk of depression and anxiety-related symptoms, as well as an increased risk of subsequent depression (20). However, previous studies have not comprehensively assessed the causal mechanisms between the consumption of UPFs and mental disorders. Although there is literature based on investigating the cross-sectional association between UPFs and mental disorders, there continues to be a relative lack of high-quality evidence on the prospective effects of UPFs as a risk factor for the development of mental disorders, as well as the pathophysiological mechanisms involved (21, 22).

Recent evidence suggests that dysregulation of lipid metabolism may play a significant role in the development of mental disorders (23–25). Furthermore, alterations in lipid metabolism and signaling resulting from UPF consumption may, in part, contribute to the pathophysiology of several important non-communicable diseases, including cardiovascular disease (CVD), type 2 diabetes, and neurodegenerative diseases (6, 25). Mental disorders and non-communicable diseases have been shown to have a strong bidirectional relationship, with several shared pathophysiological pathways mediated through common risk factors, including UPFs (26, 27). There have been several studies which have established a clear association between the constituents of UPFs and the development of several chronic diseases, including mental disorders (6, 24, 28).

Partially hydrogenated vegetable oils contain industrially produced trans-fatty acids (TFAs; commonly found in processed foods such as margarine, baked goods and deep-fried foods), which may adversely affect the blood lipid profile by raising low-density lipoprotein (LDL) cholesterol and lowering high-density lipoprotein (HDL) cholesterol, leading to increased risk of CVD (29). Increased levels of TFAs may also contribute to the pathogenesis of mental disorders as a result of altering the neuronal lipid membrane composition, leading to reduced levels of polyunsaturated fatty acid content, reduced membrane fluidity, and impaired neurotransmission (28, 30, 31). Additionally, saturated fatty acids (SFAs), which are also common in UPFs such as processed meats, fried foods and baked goods, have been linked to chronic low-grade inflammation mediated by macrophage recruitment and release of inflammatory cytokines, notably tumor necrosis factor-α (TNF-α) and interleukin-6 (IL-6), which may contribute to neuroinflammation via crossing of the blood-brain-barrier (BBB) (32). SFAs are also thought to directly induce an inflammatory response in the brain by activating toll-like receptor (TLR) receptors in microglia, causing elevated cytokines IL-6, interleukin-1β (IL-1β), and TNF-α, particularly in the hippocampus, which has been shown to correlate with depressive behavior in mice (32–34). Furthermore, high UPF intake may decrease the production of short-chain fatty acids (SCFAs) by the gut microbiota, leading to compromised BBB integrity and thus allowing more passage of inflammatory cytokines or potentially harmful toxins (e.g., titanium dioxide food colorant) (24, 35). Thus, the lipid content of UPFs may initiate inflammatory processes that converge on neuroinflammatory pathways.

UPFs have been established as an important trigger of peripheral and central inflammation (24). Inflammatory cytokines adversely affect different components of the central nervous system (CNS) and peripheral nervous system (PNS), which may, in turn, lead to degeneration of neurons and microglial cells, increased BBB permeability, as well as prolonged levels of microglial activation, which impairs neurogenesis (36, 37). These inflammatory changes may be important drivers of mental disorders as well as neurodegenerative conditions (35, 38).

Consumption of refined carbohydrates (or otherwise known as simple carbohydrates), which break down into simple sugars, may contribute to elevated levels of intracellular glucose, thereby leading to an increased production of free radicals that may damage lipids, thereby inducing lipid peroxidation and causing membrane damage (39). This has, in turn, contributed to the development of atherosclerosis, as well as contributing to increased fasting glucose levels, which increases the risk of type 2 diabetes. Furthermore, the inflammatory cytokines (IL-6, TNF-α, IL-1β) linked to UPF consumption may further contribute to elevated levels of insulin resistance as well as to the development and progression of atherosclerotic disease through both endothelial damage and dysfunction, which is mediated through various signaling pathways, including TLR, and Nod-like receptor protein 3 (NLRP3) inflammasome and nuclear factor-kappa B (NF-kB) (40–43). Insulin resistance in the brain has been linked to dopaminergic dysfunction manifesting as anxious and depressive behaviors, whilst cerebrovascular disease may potentially contribute to depressive disorders through disrupted neural connectivity and cerebral hypoperfusion (44, 45). In addition, other common additives, such as emulsifiers and stabilizing agents (e.g., carrageenan), are associated with increased levels of glucose intolerance and insulin resistance, which may, in some cases, contribute to the development of metabolic syndrome as well as mental disorders (7).

Other potential causative mechanisms may also include hormonal dysregulation due to exposure to Bisphenol A (BPA) and other contaminants found in food packaging, which, in turn, contribute to alterations to the gut microbiome, as well as other changes along the gut-brain axis (35). However, other factors, such as hyperpalatability, may contribute to hypothalamic-pituitary-adrenal (HPA) axis dysregulation, which may be implicated, in some clinical situations, in the pathological development of addictive eating behaviors via the alteration of hunger and satiety hormones (46). This drives the propensity to consume energy-dense UPFs during “emotional eating,” forming a bidirectional relationship between UPF intake and mental disorder (20, 47). Other purported mechanisms include exposure to contaminants such as advanced glycation end products (AGEs; e.g., acrylamide) formed by high-temperature industrial frying, which has been suggested to contribute to neuroinflammation and neurotoxicity mediated through the generation of free radicals, mitochondrial dysfunction and activation of glial cells such as BV-2 microglial cells, primary astrocytes, and microglia, together inducing neuronal apoptosis (8, 48–50). Nevertheless, interestingly, changes in lipid metabolism have not been systematically discussed in the context of the development of mental disorders despite a likely overlap in the pathophysiological mechanisms associated with the development of the major non-communicable diseases.

Hence, this scoping review aims to synthesize existing literature to explore the potential relationships between UPFs, lipid dysregulation, and mental disorders. Specifically, it investigates the underlying molecular pathways through which UPF consumption may disrupt lipid metabolism. Additionally, the review seeks to understand how these metabolic alterations could contribute to a range of clinical manifestations, including depression, mood and anxiety disorders, eating disorders such as bulimia nervosa, UPF addiction, attention deficit/hyperactivity disorder (ADHD), and autism spectrum disorder (ASD).

The types of constituents in the human diet vary across cultures and have evolved. However, within the context of the Western diet, UPFs now account for a growing proportion of total energy intake. At the same time, global prevalence and burden of mental disorders continue to rise exponentially, placing great strain on our health systems. There is currently a need to assess the impact of UPFs across the spectrum of mental disorders. The findings from this study may inform the development of future therapeutic interventions, clinical practices, and policymaking, both at a local level in Australia and at a global public health level.

## 2 Methodology

### 2.1 Search strategy

This scoping review was conducted in accordance with the preferred reporting items for systematic reviews and meta-analyses extended for scoping reviews (PRISMA-ScR). The search strategy was developed to capture literature examining the intersection of three core concepts: UPFs, mental health, and lipid metabolism. A comprehensive search was undertaken in PubMed, Web of Science, and EMBASE for studies published between January 2020 and July 2025 to ensure inclusion of the most recent evidence. Search strings were constructed to explore the interactions among all three concepts, associations between UPFs and mental health outcomes, as well as the links between mental health and lipid metabolism. medical subject headings (MeSH) and equivalent controlled vocabulary terms were used where applicable. Key MeSH terms included: “Food, Processed” [Mesh], “Mental Health” [Mesh], “Depressive Disorder” [Mesh], “Depression” [Mesh], “Sleep Deprivation” [Mesh], “Sleep Disorders, Circadian Rhythm” [Mesh], “Binge-Eating Disorder” [Mesh], “Anorexia Nervosa” [Mesh], “Bulimia Nervosa” [Mesh], “Food Addiction” [Mesh], “Autism Spectrum Disorder” [Mesh], “Attention Deficit Disorder with Hyperactivity” [Mesh], “Stress Disorders, Post-Traumatic” [Mesh], “Lipid Metabolism” [Mesh], “Lipid Metabolism Disorders” [Mesh].

The focus on UPFs reflects growing global concern regarding their widespread consumption and potential implications for health and wellbeing. The final database searches were completed on 18 July 2025. A manual search of reference lists and database results was also performed to ensure completeness. All identified records were imported into Covidence, where duplicates were removed, and the remaining articles were screened for eligibility.

### 2.2 Study inclusion and exclusion criteria

Studies eligible for inclusion were peer-reviewed English journal articles, reviews, or meta-analyses published between 2020 and 2025. For human studies, participants of all ages, genders, and demographics were included. Animal studies were included for mechanistic insights.

Studies were included if they compared the effect of high and low levels of UPF consumption with the risk of developing a psychiatric disorder, or if they investigated the mechanisms related to how UPFs may cause lipid dysregulation in the context of the pathophysiological mechanisms involved in the development of mental health disorders. Disorders included in the literature review were depression and anxiety, addiction-related disorder (food addiction), eating disorders (bulimia nervosa and binge-eating disorder), ADHD and ASD.

Studies were excluded if the study focused on a patient population with existing comorbidities, the intervention was not specifically UPF intake, the outcome was not the risk of mental disorder or lipid dysregulation, the setting was restricted to the COVID-19 pandemic, or the topic was not relevant to the specific psychiatric disorders included.

### 2.3 Data screening, extraction, and analysis

**Figure 1:**
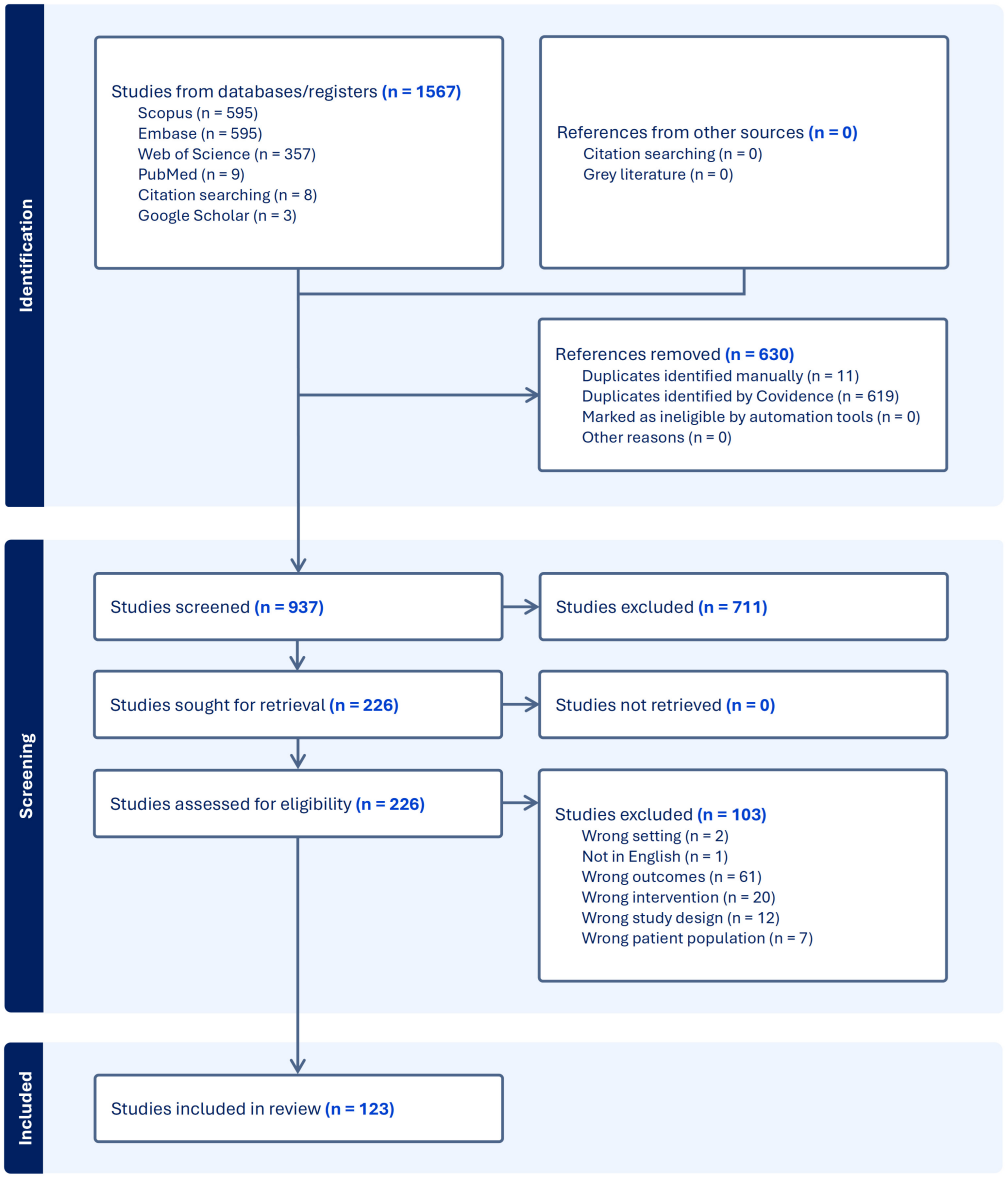
PRISMA flow diagram illustrating the screening of studies included. One hundred twenty-three studies were included in the final data extraction.

Title and abstract screening, followed by full-text review, was conducted independently in Covidence by two reviewers (CL, EP) as shown in Figure 1. All included studies were assessed for methodological rigor and risk of bias to ensure reliability. Key study characteristics, including publication year, country, study design, primary findings, and lipid measures, were extracted and summarized in a table using Microsoft Excel (see Supplementary material). Figures were created using BioRender.

### 2.4 Study quality assessment

A formal critical appraisal was not required for this scoping review. However, the study limitations were noted in the table of data extraction, and discrepancies between studies will be further explored in the Section 4 of the article.

### 2.5 Data synthesis

Data characteristics, including year, country, study type, key results, mechanisms, lipids discussed, and therapeutic implications, were extracted. Results were categorized by mental illness. Key associations were identified, and then a hypothesized mechanism to explain the associations.

## 3 Results

### 3.1 Descriptive analysis of included studies

The analysis of the included studies provides valuable insights into the field's research landscape. Two key aspects of the studies' characteristics are highlighted: the country of origin and the publication timeline.

**Figure 2:**
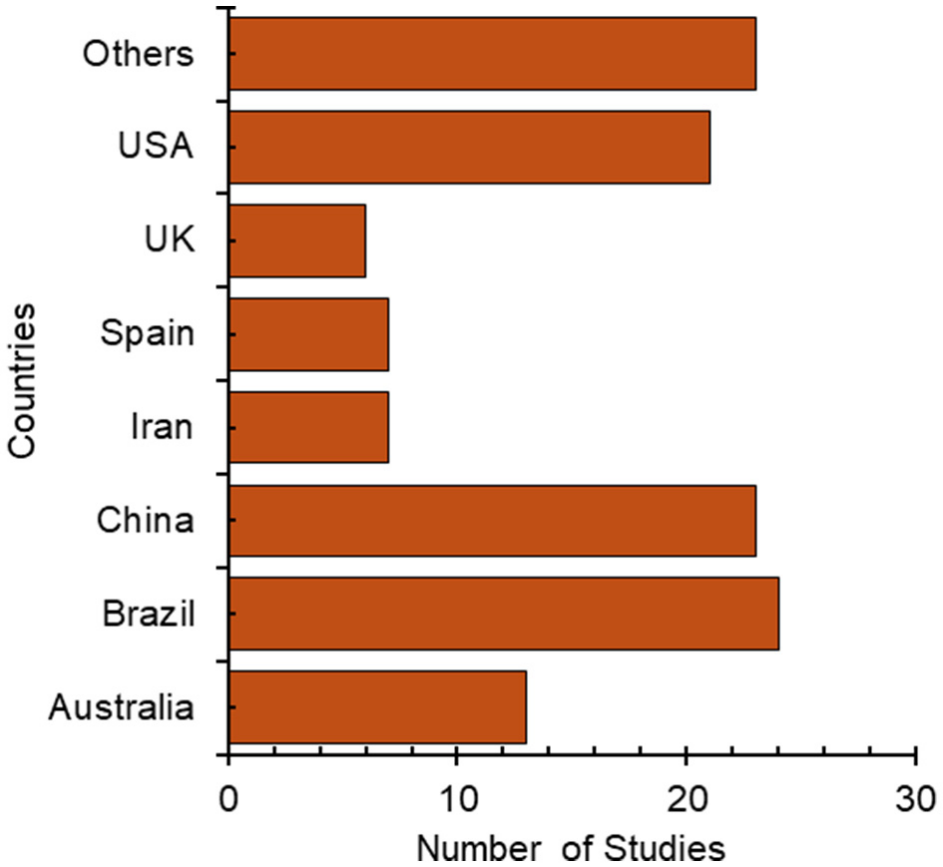
Distribution of studies by country of publication. Brazil had the most at 24 studies.

The distribution of studies across different countries, as shown in Figure 2, reveals a diverse range of research contributions. While some countries have multiple publications, others are represented scarcely and are grouped under the “Other” category. This category includes countries such as Chile, France, Germany, Iran, Italy, Japan, South Korea, Lebanon, Mexico, Norway, Saudi Arabia, Sweden, Switzerland, the Netherlands, and Türkiye. Research from these countries suggests a global interest in the topic. However, the studies' concentration in certain countries may indicate areas of focused research expertise or specific regional interests (Figure 2).

**Figure 3:**
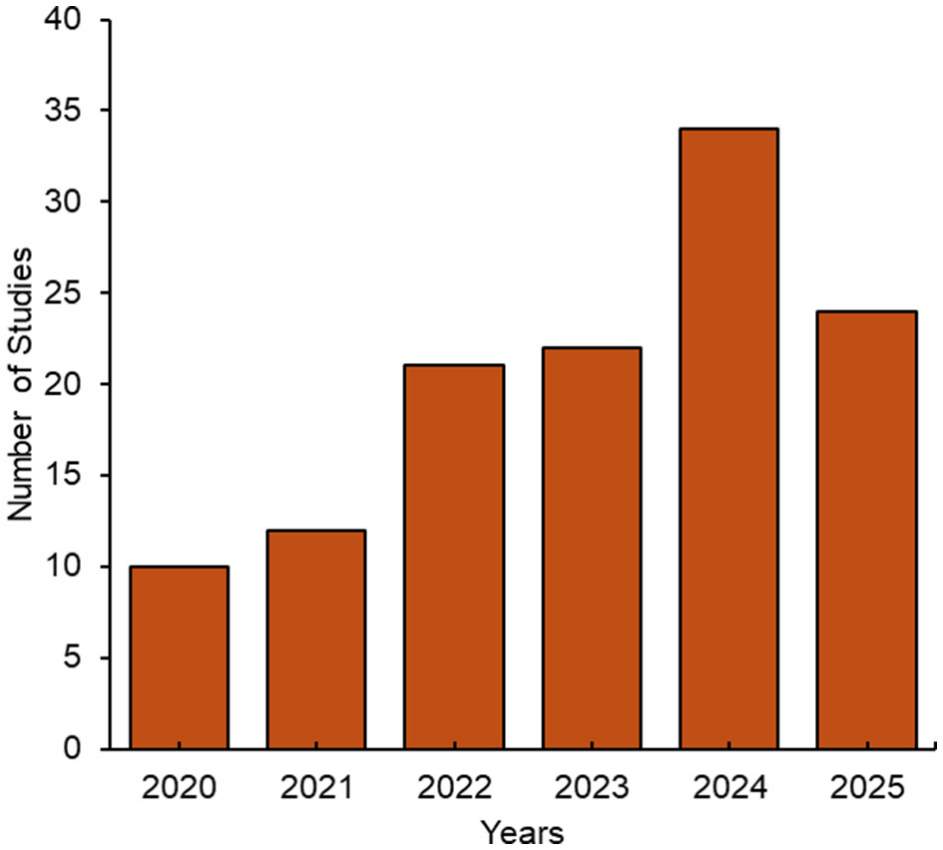
Distribution of studies by year of publication. The search was conducted in July 2025.

The temporal distribution of publications, shown in Figure 3, reveals a clear upward trend in research activity over time. Most included studies were published between 2022 and 2025, with the highest number of studies published in 2024. This surge likely reflects growing recognition of UPFs and lipid dysregulation as key mechanisms in mental health research, alongside rapid advancements in lipidomics technologies. Increased interdisciplinary collaboration between mental health and lipidomics researchers may have further contributed to the rise in scholarly output. Overall, the recent growth in publications suggests a rapidly expanding interest in this emerging field.

### 3.2 UPF and depression

#### 3.2.1 Background on known mechanisms of depression

One of the most widely recognized pathophysiological mechanisms involved in the development of mood disorders, such as depression, is the monoamine hypothesis (51). This hypothesis suggests that there are reduced levels of key neurotransmitters, including serotonin, dopamine, and noradrenaline, within the brain that contribute to both the onset and persistence of depressive symptoms (51, 52). This forms the basis of tri-cyclic antidepressants and monoamine oxidase inhibitors in depression therapy, as these drugs increase the levels of the monoamines within the brain, and relieve depression symptoms (51). Alternative hypotheses exist, such as a model involving the HPA axis (53). Here, the HPA axis becomes overactive in response to stressful events, resulting in increased glucocorticoid and cortisol levels (53). Increased glucocorticoids can also result from inflammation in the brain (53).

Another model is the stress-diathesis theory, which suggests that stressful events during life can alter neuroplasticity and transmission, which leads to the symptoms of major depression (54). Gray matter volume changes are different in healthy patients compared to those with MDD (55). Patients who reported higher stressful life events had an increased risk of depression (56).

Depression risk is also a combination of genetic and environmental factors (57, 58). This was supported since first-degree relatives of patients with MDD have a 2.8 times higher risk of MDD (57). Together, these findings suggest that depression is a multifactorial disorder involving complex neurobiological pathways. This section will explore the various mechanisms involved in depression, providing a more comprehensive understanding of its underlying pathophysiology.

#### 3.2.2 Implications of UPF consumption on depression risk

Previous studies have demonstrated a significant association between UPF and depression, which remains after adjusting for a range of confounders (46, 59–62) (Table 1). However, in some studies, no significant association was found between UPF and depression (60, 63, 64). The specific food types that were investigated as part of these studies included sugar-sweetened beverages (SSBs) (61, 65–69), although there was a stronger association between males and the intake of SSBs than among females (65). A dose-dependent relationship was also found with energy drinks (69). Also, fast foods and fried foods (67, 70), processed meat (71), smoked food (70), and foods containing higher levels of sodium (71) were associated with an elevated lifetime risk of depression. However, further studies are needed to establish whether there is a causal relationship between the intake of UPF and the risk of depression. Some studies have identified a higher lifetime depression risk among females compared to males (72, 73). In contrast, in another study, it was found that there was a higher risk of depression among males who were under 60 years old (74). This may arise due to hormonal or metabolic differences across the sexes. Associations were found globally, including in Korea, Brazil, China, Australia, Spain, and France (21, 75–79). Notably, individuals in the highest quartile of UPF intake had a significantly greater risk of depression (35, 62, 78, 80, 81). Additional evidence demonstrates a dose-dependent relationship, in which increasing UPF consumption corresponds to a progressively higher risk of depression (22, 25, 66, 82–84). In addition, UPF consumption during pregnancy was associated with an increased odds of depression symptoms in offspring compared to healthy non-clinical controls (85).

**Table 1:** Summary of studies implicating increased UPF consumption and dysregulated lipid metabolism associated with depression or anxiety.

**Lipid subtypes implicated**	**Mechanisms**	**Key findings**	**Therapeutic implications**	**Source**
**Depression**				
Saturated fatty acids Trans-fatty acids Monounsaturated fatty acids Polyunsaturated fatty acids Omega-3 fatty acids Omega-6 fatty acids cholesterol	– UPFs contain sugar, sodium, saturated fats, and TFAs, with reduced intake of whole foods. These have pro-inflammatory properties. – Food processing agents such as additives, emulsifiers, preservatives–e.g., TiO_2_, induce inflammation and oxidative stress, emulsifier carboxymethylcellulose, and polysorbate-80 disrupt microbiota-inducing inflammation	– High UPF intake (33% of daily intake) significantly increased likelihood of recurrent depressive symptoms – Benefits of “healthy diet,” such as Mediterranean diet, are attenuated if there is also significant UPF intake	–	(31)
Saturated fatty acids	– Similar to LPS, SFAs (e.g., palmitic, lauric, stearic acid) activate TLR4 on macrophages and microglia–> phosphorylation of the Ikappa B alpha (IkBa) protein and disinhibition of NF-kB signaling -> synthesis of pro-inflammatory cytokines (IL-1β, IL-6, TNF-α, CRP–inflammatory hypothesis of depression). – SFAs can raise the ratio of gram-negative gut bacteria and increase gut permeability -> LPS leakage into bloodstream, causing inflammation (metabolic endotoxemia). – SFAs cross the BBB and bind to TLRs on resident microglia -> proinflammatory response (hypothalamus and NTS) -> alter neural circuits regulating energy balance, leading to excess nutrient consumption, impairing mood regulation/reward processing. This response precedes systemic inflammation; however, chronic SFA consumption can induce inflammation in other brain regions, leading to learning and memory impairment.	– Mechanisms identified	– Emphasizes whole food diet and shift to preventative chronic disease management – Highlights need for further studies on neuroinflammation e.g., advanced imaging techniques (35)	(35)
– Saturated fatty acids – Trans fatty acids	– High UPF intake → poor nutrient profile high saturated/trans fats, sugars, additives; low antioxidants/fiber -> alters gut microbiota → dysbiosis → neuroinflammation via gut-brain axis. – Increased systemic inflammation (IL-6, TNF-a, leptin) -> HPA axis activation -> increased depressive symptoms	– From the Brazilian Longitudinal Study of Adult Health, higher UPF consumption is associated with a higher risk of persistent depression and incident depression over 8 years. Odds of persistent depression increased across quartiles of UPF consumption. – Substituting UPF with minimally processed foods reduces depression incidence	– Encourage substitution of UPFs with unprocessed/minimally processed foods	(105)
– Saturated fatty acids – Omega-3 – Polyunsaturated fatty acids – Monounsaturated fatty acids	– Saturated fatty acids → ↑ IL-6 secretion → inflammation; positively associated with CRP, IL-6, sVCAM, sICAM. – Omega-3 fatty acids → inhibit inflammatory response. – High sodium → ↑ TNF-α. – Vitamins A, C, E, β-carotene (often low in UPFs) → ↓ CRP and IL-6. – UPFs lack antioxidants, folate, and fiber → reduced anti-inflammatory effects and impaired gut microbiota → dysregulated cytokine production.	– Chronic low-grade inflammation-elevated levels of biomarkers e.g., CRP, IL-6, and TNF-α–is a risk factor for neuropsychiatric disorders, including depression and ADHD – Western dietary patterns higher in UPFs are associated with high levels of inflammatory biomarkers – Study from Oddy et al. (190) -> western diet from 14–17 years of age associated with higher hs-CRP levels, which correlated with increased depression risk	Potential for healthy dietary patterns (e.g., Mediterranean, DASH, low-GI diets) to reduce inflammation	(172)
– Saturated fats – Trans fats	– High UPF intake → increased refined sugars (such as high-fructose corn syrup), saturated/trans fats → oxidative stress, mitochondrial dysfunction, and activation of pro-inflammatory pathways (NF-κB, mTOR, JNK, AKT). – Saturated and trans fatty acids damage the endoplasmic reticulum, alter cellular membranes. – Low fiber and high UPF intake → gut microbiota dysbiosis → reduced SCFAs, increased LPS-producing bacteria, intestinal barrier dysfunction -> bacterial translocation into bloodstream → systemic inflammation → altered neurotransmission. – Toxic by-products such as acrylamide, acrolein, polycyclic aromatic hydrocarbons, and furan are shown to be neurotoxic and cause inflammasome-related neuroinflammation	– Highest quartile of UPF intake -> higher odds of having depressive symptoms in younger adults 18–35 years	–	(80)
– Saturated fat	– Aspartame artificial sweetener, inhibits the synthesis and release of neurotransmitters, dopamine, norepinephrine, and serotonin, which are important in depression development – Macronutritional characteristics of UPFs (rich in saturated fats and sugar, and low in dietary fiber, micronutrients, and phytochemicals) influence a variety of interacting pathways, including inflammation and oxidative stress	– High UPF -> increased risk of depression and combined depression anxiety (as common mental disorders) with highly suggestive evidence	– Promote dietary patterns low in UPFs and rich in minimally processed foods–Implement public health policies such as food labeling and price adjustments. – Encourage consumer awareness and corporate responsibility to shift toward healthier food production. – Call for large-scale cohort and intervention studies to establish a safe recommended daily intake (RDI) of UPFs.	(102)
– Omega-6 – Omega-3 (EPA and DHA)	– Western diet (processed meats, sugary drinks, take-away foods, snacks) → proinflammatory; high omega-6/omega-3 ratio → ↑ pro-inflammatory eicosanoids, ↓ BDNF, ↓ membrane fluidity → impaired neuronal signaling. – Omega-3 normally protective: DHA abundant in brain → ↓ proinflammatory cytokines, ↑ BDNF; EPA → anti-inflammatory via ↓ TNF-α, IL-6, IL-1β through NF-κB inhibition. – Mg and Zn deficiency in Western diet → impaired neuroplasticity → reduced BDNF production.	– Western dietary pattern high in UPFs is positively associated with depressive symptoms	–	(43)
– Omega-3 PUFAs (EPA and DHA)	– Bidirectional relationship between excessive energy intake and depression – UPF intake causes systemic inflammation with increased levels of inflammatory cytokines (IL-6, TNF-α, IFN-β, and complement C5) in the CSF-> neuroinflammation -> decreased production of monoamine neurotransmitters such as dopamine – Overactivation of the HPA axis – Dysfunction of BDNF – EPA and DHA (omega-3 PUFAs) from fish have anti-inflammatory effects -> found to have an inverse relationship with the level of inflammatory markers and depression – UPF may impair production of SCFAs (e.g., butyrate), which enhance the integrity of the BBB. Decrease SCFA -> increased BBB permeability -> neuroinflammation	– High UPF consumption -> increased risk of depression	– Limit UPF intake and promote traditional diets (e.g., Mediterranean, Japanese). – Encourage intake of fish, vegetables, whole grains, and fiber. – Supplement Ω3 PUFAs, vitamins (D, folate), and minerals (iron, zinc) if deficient – Avoid using antidepressants that cause weight gain	(89)
– Omega-3	– Associations between MC4R (melanocortin) gene, CRY1 (circadian regulation) gene, Cav_1 with depression – A synergistic association between genetic predisposition and UPF intake may be attributed to the displacement of antioxidants from fruit/veg. Also, folate deficiency can impair serotonin synthesis and CNS function. – Omega-3 in fish is protective against depression	– Study found higher UPF intake -> increased odds of depression and anxiety; however association was NOT significant – Higher UPF intake -> higher DASS-21 scores – Significant interaction between UPF intake + genetic risk score (based on MC4R, CAV1, CRY1 polymorphisms) on depression and DASS -> suggests that UPF combined with genetic predisposition has a synergistic effect on risk of depression or mental illness	–	(60)
– Lipoproteins	– High intake of fats, calories, sugar, salt, combined with low micronutrients and fiber -> development of disease – Processing such as heat treatment, food additives and packaging generate carcinogenicity and genotoxicity – Changes in lipoprotein profiles	– Two cohort studies found consistent positive associations between high UPF consumption and increased risk of depression. – The highest quartile of UPF intake is significantly associated with a greater incidence of depressive symptoms	– Reduce UPF consumption and promote minimally processed foods – Food taxation and marketing regulation to limit UPF exposure. – Dietary guidelines should reflect the risks of UPFs and encourage whole-food-based diets. – Public health campaigns to raise awareness of UPF-related risks.	(75)
– Lipids in general–modified into AGEs via glycation	– AGEs are glycated proteins or lipids that bind to the receptor for advanced glycation end products (RAGE), which mediates various inflammatory pathways promoting ROS. Dietary AGEs are formed during prolonged high-temperature cooking such as frying, roasting or grilling (Maillard reaction) – Dietary AGE stimulates inflammatory response via innate immune cells -> interact with receptor on BBB -> free radicals, activation of NF-κB, and production of pro-inflammatory cytokines -> cellular, mitochondrial dysfunction, intracellular ROS, and apoptosis – RAGE activation -> glial activation, cytokine and ROS production -> neuroinflammation. Excess RAGE signaling downregulates normal detoxification pathways – AGE impairs the BDNF-TrkB signaling pathway -> implicated in depression – AGE causes endothelial dysfunction by the formation of cross-links in the arterial wall and collagen -> stiffness -> impaired microcirculation in the brain	– UPF associated with high levels of (AGEs) -> strong association with increased risk and severity of MDD. Mild associated also found for anxiety, but not significant – AGEs mediated the mortality risk of major depression	– Low-AGE diets (e.g., Mediterranean, DASH) – Cooking modifications: avoid dry heat (frying, grilling), use moist heat (boiling, steaming) – Nutritional interventions: antioxidants (e.g., flavonoids, thiamine, pyridoxamine) – Monitoring AGE: RAGE ratio as a biomarker	(8)
– Lipids in general	– Protective effects of minimally processed foods may be attributed to essential micronutrients (B vitamins, vitamin D, and zinc), which contribute to brain health and help reduce the prevalence of depression and anxiety – Antioxidants, fiber, and micronutrients improve inflammation, decrease oxidative stress, and balance the gut microbiome – Bidirectional relationship between diet and mental health -> poor mental health may lead to increased UPF consumption -> feedback loop	– Higher energy and lipid intake from UPF -> more depressive symptoms – Women with depressive symptoms consumed less UMPF (unprocessed and minimally processed food). Depressive symptoms accounted for 7.6% of lower UMPF intake – Higher UMPF and UMPF lipid intake -> less depression and anxiety symptoms	– Professional dietary advice and treatment from a multidisciplinary team, including physicians, psychologists, and nutritionists	(82)
– HDL cholesterol – LDL cholesterol – Triglycerides	– Alteration of serum lipid concentrations, modified gut microbiota and host–microbiota interactions, obesity, inflammation, and insulin resistance	– Higher UPF score -> more likely to have depression according to the CESD-10 scale (19 vs. 14.9%) – Higher UPF intake: total cholesterol decreased, and triglycerides increased slightly	– Promote Mediterranean Diet – Consider socioeconomic and lifestyle factors that may limit access to unprocessed foods	(77)
–	– Women are more affected by UPF, potentially due to different hormonal and metabolic profiles, as well as different microbiota composition, which could impact the gut-brain axis	– UPF is significantly associated with the cross-sectional increased risk of depression in both males and females aged 18–34 years. The risk was increased in older women aged 35–54 years or over 55 years, with an odds ratio of 1.41. A significant association was not found in older men. – Higher adherence to a healthy dietary pattern was associated with lower depression risk in women aged 18–54 years.	– Higher adherence to the Mediterranean diet reduces depression risk – Suggestion for depression treatment guidelines to be informed by age and sex, with a greater focus on nutrition in women	(21)
–	–	– Women with a high level of adherence to a healthy diet (Brazilian food guide golden rule) had reduced risk of depression. Hence, UPF consumption is associated with an increased risk of depression, and minimally processed foods are protective.	– Recommends adhering to healthy diet guidelines	(173)
–	– Sugar-sweetened beverages (SSBs) contain high fructose, which may increase corticosterone levels and dysregulate the hypothalamic–pituitary–adrenal (HPA) axis – SSBs increase the secretion of proinflammatory cytokines, contributing to depression – Artificial sweeteners (e.g., aspartame, sucralose) may cause glucose intolerance via intestinal dysbiosis – Conversely, natural juices contain various vitamins, carotenoids, flavonoids and other bioactive compounds that have beneficial effects on depression	– Higher consumption of SSBs -> 26% increase in risk of depression – Artificially sweetened beverages showed increased risk of developing depression, >2 units/day -> 40% increased risk – Moderate consumption of natural juices (NJs) is associated with a lower risk of depression	– Replacing SSBs and ASBs with NJs may reduce depression risk. – NJs may serve as a healthier alternative to sugary beverages. – Support dietary recommendations to reduce SSB and ASB intake	(110)
–	– Prolonged exposure to inflammatory cytokines -> dysfunction of monoamine neurotransmitters such as NA and dopamine -> affects gut microbiome, contributing to depression. Normally, dietary fiber is broken into SCFAs, which regulate inflammation and produce γ-aminobutyric acid and serotonin -> reducing depression – Lack of healthy nutrients which normally have a positive impact on mental health: Omega-3 fatty acids, phospholipids, cholesterol, niacin, folate, vitamin B6, vitamin B12, and antioxidant vitamins. Low tryptophan intake may impair serotonin synthesis -> anxiety and depression – Excessive consumption of added sugars can disrupt the gut microbial environment, -> inflammatory responses, insulin resistance, oxidative stress, endorphin and dopamine dysregulation -> depression	– Higher consumption of UPFs -> 1.85 fold higher cross-sectional risk of depression in middle-aged Korean women – Highest healthy diet scores -> 0.56 fold lower risk of depression	– Promote public education to increase intake of fruits, vegetables, and whole grains. – Implement policies to encourage consumption of fiber-rich foods.	(76)
–	– ↓ Mesocorticolimbic volumes (amygdala, cingulate cortex, ventral putamen, dorsomedial frontal cortex) → impaired reward processing and motivation. – UPF components (nanoparticles, trans fats, BPA) → disrupt amygdala–hippocampal function. – ↓ Dorsomedial frontal cortex activity → weaker impulse control and resistance to immediate rewards. – Altered ventral putamen/amygdala connectivity → ↑ food craving and disrupted reward signaling (seen in overweight individuals). – Changes mirror depression-related blunted reward responses. – ↑ WBC → inflammation-mediated link between UPF intake and depressive symptoms.	– ↑ UPF consumption -> ↑ depressive symptoms; however, lost significance after adjusting for education – ↑ UPF -> lower volumes of ventral posterior cingulate cortex and left amygdala (significant after adjusting) – ↑ UPF -> significant positive association with WBC levels -> mediated association between UPF and depressive symptoms – Patients with obesity showed decreased volume of the putamen and the dorsomedial frontal cortex	– Further research is needed for the adverse effects of prolonged UPF consumption during late childhood and adolescence, as these are key neurodevelopmental periods that coincide when the higher UPF consumption described	(30)
–	– High UPF intake reduces brain volume (putamen, amygdala, frontal cortex) and increases inflammation (↑WBC). – CAF diets in animals cause neuroinflammation, oxidative stress, anxiety-like behavior, neurotransmitter disruption, and BBB damage. – Depression linked to ↑CRP, ↓SCFAs, and gut dysbiosis (↑Escherichia, Shigella, ↓Trp, KYN, butyrate). – Oxidative stress causes DNA damage and telomere shortening, linking depression to aging and chronic disease. – RecQ dysfunction contributes to depressive-like behavior and neurodegeneration	– High UPF -> increased risk of depression and depressive symptoms across multiple cohorts [NutriNet-Santé cohort, NutriNet-Brasil cohort, SUN Project (Spain)]	– Implement public health strategies to minimize UPF consumption, promote minimally processed foods – Advises for collaboration between policymakers, healthcare professionals, and the food industry to develop dietary guidelines	(95)
–	– High energy density, high sugar and fat content, low satiety, additives, gut microbiota disruption, and chemical contaminants (e.g., endocrine disruptors)	– Two large cohort studies (NutriNet Santé and SUN) found that higher UPF consumption was associated with increased risk of depression – One cross-sectional study (Brazil) found that higher UPF intake was associated with greater odds of depressive symptoms during pregnancy	– Reduce UPF consumption at the population level; adopt the NOVA classification in dietary guidelines – Promote fresh or minimally processed foods–Address marketing, portion size, and accessibility of UPF; regulate additives and packaging chemicals	(85)
–	– Factors mediating the diet-depression association: oxidative and antioxidant defense systems, brain plasticity, microbiota-gut-brain axis, mitochondrial dysfunction, tryptophan kynurenine metabolism, neurogenesis, and epigenetics	– Depression -> higher consumption of UPF among women	– Women represent a greater global prevalence of depression, and seem more susceptible to the diet-depression association -> target women in public health interventions – Implement clear dietary guidelines – Apply taxes on processed foods and offer financial incentives for whole foods	(174)
–	– Poor nutritional profile and displacement of nutritious foods – Food additives, gut microflora dysbiosis and increased intestinal permeability increase inflammatory response – Reduced satiety signaling due to altered physical properties and destruction of food matrix caused by ultra-processing of foods	– Two prospective cohort studies (France and Spain) showed that the highest quartile of UPF intake -> significantly higher risk of subsequent depression (increase in risk of 31% over 5 years and 33% over 10 years)	– Incorporating the concept of ultra-processing into dietary guidelines – Applying NOVA classification in food labeling and food procurement policies	(1)
–	– Food additives and artificial sweeteners lead to changes associated with poor mental health: impaired glucose tolerance, increased inflammatory mediators, oxidative stress, neuroinflammation, neuronal mitochondrial dysfunction, alteration in tryptophan metabolism, altered HPA axis, changes in local expression of neurotrophic growth factors	– Highest level of UPF intake -> higher odds of having mild depression (OR 1.81), more anxious days, more mentally unhealthy days	–	(83)
–	Discusses mechanisms related to other NCDs that may overlap with mental disorders: – Energy density -> overfeeding -> metabolic dysfunction – Rapid gastric emptying of refined carbs alters gut-brain signaling via GLP-1, GIP, and CCK, which alters satiety and appetite regulation – Refined carbohydrates accelerate endothelial dysfunction and promote oxidative stress via the formation of AGEs – Disruption of food matrix damaging to gut microbiota -> impacts nutrient bioavailability, digestion kinetics, glycaemic control and satiety – Acellular nutrients, PAMPs and artificial additives promote inflammation via altered gut microbiota	– NutriNet-Sante cohort showed increased UPF -> 21% increased risk of incident depressive symptoms – SUN cohort shows the highest quartile of UPF intake -> 33% increased risk of developing depression	– Encourages Mediterranean diet, DASH diet – Emphasizes the need for research into food additives, texture and food matrix integrity – Suggests implementation of policies: banning or reduction of specific substances or processing methods, improved food labeling, improved availability and affordability of minimally processed foods	(109)
–	– Displacement of nutrients – Hypercaloric and less satiating, leading to excessive energy intake, potentially mediated through lower levels of appetite-suppressing hormones (e.g., peptide YY) – Destruction of the food matrix alters bioavailability and gut microbiome – Additives may promote inflammation and cause metabolic dysregulation	– Results from NutriNet Sante France and SUN Spanish cohort studies	–	(101)
–	Highly caffeinated foods cause anxiety and nervousness, which stimulates emotions. Short-term relief from fatigue followed by long-term emotional decline Possible association with SES	– Energy drinks were associated with depression; increased frequency had a higher association with depression		(69)
–	–	SUN project -> higher UPF intake significantly associated with increased risk of depression -> pooled hazard ratio 1.22 means 22% higher risk	– Recommends research in the form of longitudinal studies, and the need for predefined cut-off ranges for UPF consumption rather than “quartiles” to homogenize data across countries	(6)
–	– Disrupted food matrix leads to rapid absorption of sugars (e.g., fructose), which stimulates the brain's dopaminergic reward system, potentially contributing to overconsumption and mood dysregulation	– Convincing high-grade evidence that greater SSB consumption -> 31% higher risk of depression (drawn from Hu (191) meta-analysis)	– Recommends limiting sugar intake to ideally < 5% of daily energy consumption – Incorporate avoidance of SSBs into national dietary guidelines and policies, implement sweetened beverage taxation, or warning labels on packaging	(163)
–	– Possible reverse causality despite prospective design: development of depression may have contributed to increased UPF intake as low mood -> type 3 serotonin receptors (5-HT3), which are present in all mesolimbic circuits, induce food consumption in response to hunger and palatable and rewarding properties of the food – Elevated TNF, an inflammatory and neurotoxic cytokine, is associated with degeneration of the myelin sheath	– Highest quartile of UPF consumption -> higher risk of developing incident depression after follow-up (82% increase)	– A prospective longitudinal study still cannot exclude the possibility of bidirectional causality	(62)
–	Deficiency in micronutrients due to the absence of whole foods, also gut microbiota imbalance pro-inflammation by UPF	Higher UPF intake and depression for females		(72)
–	Impacts on neurochemistry and neuroinflammation	Daily UPF consumption is associated with a higher depression score	Depression is associated with poorer sleep	(175)
–	– Chronic inflammation related to UPF ingredients	High UPF >4 servings/day –> increased risk of depression ^**^Significant for females in subgroup analysis, and with a higher BMI >30	–	(73)
–	–	Increased UPF dose vs. Depression^**^, UPF vs. Depression^**^ OR: 1.01 (1.00–1.01), relationship maintained in multivariable logistic regression	–	(25)
–	–	– Increasing dose of UPF –> increased risk of depression (using strict and broad depression definition). UPF components vs. Depression identified: artificial sweeteners and beverages had a highly significant OR – Reducing UPF intake by at least three servings/day lowered depression risk	UPF increases depression risk	(66)
–	–	Fried foods not associated w/depression	UPF is not associated with depression risk	(64)
–	Proposed metabolic dysfunction through inflammation, emulsifiers	Variation between papers in the relation between SSBs and depression, majority found associations between UPF and depression	–	(108)
–	– UPF have a worse nutrient profile, and added sugar worsens the glycaemic index, causing peaks in blood glucose levels. Compensatory responses could be the secretion of cortisol, adrenaline, which are associated with lower mood – Inflammation is associated with depression	Each 10% increase in dietary share of UPF was associated with a 10% increase in depression symptom risk UPF vs. depression HR 1.32 (1.19–1.46)	–	(84)
–	– Sugar → ↓ BDNF → impaired neurogenesis and hippocampal atrophy → ↑ depression risk. – ↑ CRP and IL-6 → proinflammation → altered neurotransmitters affecting mood.	Chinese cohort: Quartile 4 significant HR 1.2 for animal food, and 1.22 for sugar-rich pattern against depressive symptoms The traditional dietary pattern was protective of depression UK cohort: UPF vs. depression, significant 1.39 HR	Consider healthy traditional diets to prevent mental illness UPF vs. depression supported	(92)
–	– Proinflammatory signals → activate innate immune system → low-grade inflammation → impaired neuronal function and synaptic plasticity → mental disorders. – ↑ CRP, IL-6, IL-17, oxidative stress markers; ↓ BDNF. – Antioxidants and anti-inflammatories → modulate gut microbiota → reduce depression risk.	Inflammatory food pattern (significant) and processed foods pattern associated with depression Healthier diets (vegetables and fruits, animal foods) reduce risks	UPF vs. depression supported	(93)
–	– UPFs → lower diet quality → ↑ risk of depression. – Food additives and artificial sweeteners → may ↑ beta-theta brain wave ratio → associated with negative emotions.	US cohort: UPF quartile 4, significant OR 1.34 (1.00–1.78) for depressive symptoms	UPF vs. depression supported	(81)
	– Inadequate micronutrient intake – Dysregulation of systems common across both cardiometabolic and depressive disorders: HPA axis hyperactivity, chronic low-grade inflammation, leptin and insulin resistance, microbiota-gut-brain axis dysfunction and reduced BDNF – Processing also impairs the brain's ability to gauge the nutritional content of food -> UPF susceptible to overeating and also lead to metabolic dysfunction – Exercise may attenuate some effects of UPF (anti-inflammatory and beneficial neurochemical effects)	– Highest quartile of UPF intake -> higher risk of developing subsequent depression – Association was stronger for individuals with lower levels of physical activity	–	(78)
**Anxiety**				
Triglyceride (as a marker for metabolic dysfunction contributing to systemic inflammation)	– Neurotransmitter dysregulation: CAF diet reduced dopamine and serotonin in the prefrontal cortex, impairing reward and mood regulation; COMT enzyme expression increased, accelerating dopamine degradation – Gut dysbiosis -> reduced SCFA production -> increased gut permeability -> elevated LPS and systemic inflammation activated neuroinflammatory pathways, increasing IL-6 in the brain and compromising BBB integrity	– Cafeteria diet (CAF) rich in UPFs caused significant metabolic and neurological changes in aged rats, including: increased IL-6 expression in the prefrontal cortex and reduced blood-brain barrier integrity (lower CLDN5 protein), altered neurotransmitter levels: decreased dopamine and serotonin, reduced glutamate, Increased anxiety-like behaviors in open field and elevated plus maze tests, elevated plasma (LPS), indicating metabolic endotoxemia – CAF diet also significantly increased triglycerides – Switching to a standard diet after CAF diet also induced anxiety-like behavior, suggesting withdrawal-like symptoms – Calorie restriction (CR) after CAF mitigated many of these effects, including reduced anxiety-like behavior and improved metabolic and gut microbiota profiles	– Promotes calorie restriction as a promising strategy to mitigate anxiety symptoms in rats on CAF diet -> potential translational implications for humans on high UPF diet	(116)
–	– Food additives e.g., aspartame, polysorbate 80 (P80) and carboxymethyl cellulose (CMC) shown to cause anxiety-like behavior in rats – Bidirectional relationship between UPF and anxiety	– University students who consumed fewer types of UPF had lower odds of anxiety	– Health education prioritized, especially about UPFs and a healthy diet	(176)
–	– Anorexigenic brain circuits are particularly sensitive to emulsifiers during pregnancy. – Emulsifiers can affect the development of hypothalamic neurocircuits in early life. – Maternal consumption of emulsifiers → gut dysbiosis in offspring → predisposition to colitis and potential psychological effects.	Males were more sensitive to neuropsychological disruption	Better nutritional advice is required during gestation	(177)
–	– DHA/EPA effects could be by inhibiting the activation of phospholipase A2, which goes on to form proinflammatory products	DHA/EPA reverses the consequences of obesity in the brain CAF diet (processed) increased triglyceride levels In liver: TNF-α were increased following CAF, but was reversed by DHA/EPA, IL-6 was unaffected following CAF CAF increased anxiety in mice, which was reduced following DHA/EPA administration	Inflammation caused by processed foods can be reversible with anti-inflammatory administration	(117)
**Depression and anxiety**				
Glycerolipids 61 lyso- Phosphatidylcholines (LPC) Phosphatidylcholines (PC) Arachidonic acid	– Acrylamide exposure causes anxiety- and depression-like behavior via disrupted cerebral lipid metabolism and neuroinflammation. – Chronic exposure damages the blood–brain barrier and activates inflammatory and immune responses. – Lipid markers (LPC, PC) indicate inflammatory storms in the brain (seen in zebrafish). – Alters PPARγ and JAK-STAT signaling, lipid transport, amino acid, linoleic and arachidonic acid metabolism. – Leads to oxidative stress (increased ROS, lipid peroxidation) and impaired brain function.	UPF is significantly associated with depression and anxiety (individually) Higher risks were observed for males and younger age (< 60) for depression and anxiety These were independent of other diseases	Fried food and fried potato consumption are considered for humans	(74)
Ceramide, sphingomyelin	– TIOO → alters microglial differentiation and inflammatory factor levels → astrocyte activation → disrupted glutamate circulation. – M1 microglia ↑ IL-1β, IL-6, TNF-α, NOS2 → pro-inflammatory response. – M2 microglia ↓ IL-4, IL-10 → reduced neuroprotection. – Astrocytes → ↑ proliferation, GFAP precipitation → astrocyte damage. – ↓ BDNF secretion and ↓ glutamate receptor function → disrupted glutamate–glutamine cycle → exacerbates neuronal damage.	TiO_2_-treated mice exhibited depression- and anxiety-like behaviors, including reduced food intake and increased activity in the elevated plus maze. These mice also exhibited abnormal microglial function, characterized by altered differentiation and the release of inflammatory factors. Levels of ceramide and sphingomyelin differed from those of controls, and GLT1 expression was decreased, leading to impaired glutamate–glutamine cycling.	Long-term intake of thermal oxidized oil leads to anxiety and an inclination toward depression in mice	(86)
– Triglycerides – Cholesterol – LDL cholesterol	– Grain-fed rats → ↓ BDNF and NT-3 in hippocampus and prefrontal cortex → depression and cognitive decline. – ↓ Serotonin, dopamine, noradrenaline → supports monoamine hypothesis of depression. – ↑ MDA and ↓ GSH → oxidative stress → lipid peroxidation → ↓ membrane fluidity → altered neuronal permeability and impaired neurotransmitter binding. – Hyperglycaemia → ↑ AGEs → ↑ free radicals and oxidative stress → damages lipids, proteins, DNA. – Hyperglycaemia-induced *de novo* lipogenesis → ↑ triglycerides, cholesterol, LDL → systemic inflammation and oxidative stress → impaired neuronal function.	– In rats: whole or refined grain diet -> anxiety-like and depressive-like behaviors manifesting as increased immobility in the Forced Swimming Test (FST) and preference for closed arms in the Elevated Plus Maze (EPM), indicating heightened anxiety and despair	–	(52)
– Omega-6 PUFA	– UPFs → systemic inflammation, oxidative stress, poor nutrition → ↓ tryptophan → ↓ serotonin → depression. – High omega-6 fats in UPFs → ↑ proinflammatory eicosanoids → ↓ BDNF, impaired synaptic/cognitive function, altered neuronal membrane fluidity. – High sugar → endothelial dysfunction, inflammation, ↑ insulin → negative mood effects. – Stress/mental illness → HPA axis dysregulation → ↑ glucocorticoids → insulin resistance, altered appetite hormones → ↑ UPF consumption. – Depression → reduced motivation to eat healthy → reliance on calorie-dense UPFs.	– Cross-sectional studies showed junk food consumption increases the odds of having stress and depression – Pooling of cohort studies showed junk food consumption significantly increases the odds of depression – Analysis of studies relating to anxiety showed UPF consumption associated with increased anxiety symptoms	–	(46)
–	–	UPF vs. depression and anxiety (individually) supported with multivariable analysis	UPF vs. depression and anxiety (individually) supported	(166)
–	– Reverse causation: depressed individuals may have greater sweet cravings – SSBs -> cardiometabolic dysregulation, metabolic syndrome -> risk factors for mental illness – SSBs induce chronic inflammation -> depression and anxiety	– SSBs are associated with an increased risk of depression in men – Artificial sweetened beverages are associated with depression in women – Artificial juice linked to higher anxiety in men	– Recommendations and policies to reduce consumption of soft drinks/SSBs to prevent mental comorbidities	(115)
–	– Poor nutritional quality and displacement of nutrient-rich foods – Use of additives and contaminants from processing	– Significant but low certainty evidence for UPF -> depression and anxiety	– Dietary guidelines and public health policies to limit UPF consumption, emphasize the need for clearer classification systems (beyond NOVA)	(178)
–	– Poor nutritional quality (low fiber, vitamins; high sugar, salt, fats) – Harmful additives (e.g., artificial sweeteners, emulsifiers) – Industrial by-products (e.g., furans, heterocyclic amines, acrolein, acrylamide, and advanced glycation end products) – Packaging contact material (bisphenols and phthalates) – All can interfere with cell signaling pathways involved in glucose homeostasis and alter gut microbiota/gut barrier function -> inflammation and metabolic change – Alteration of the food matrix may affect digestion, nutrient absorption and satiety – Addictive qualities may stem from rapidly absorbed carbs and high fat	– Higher UPF intake -> increased risk of common mental disorders (CMDs) (broadly defined as a CIS-R score of >12) – Different subgroups of UPFs had differing effects: sweet snacks, ready-to-eat/heat-mixed dishes, and sweetened beverages increased risk -> CMDs, higher intake of sweetened beverages and non-dairy sweet snacks/desserts increased risk -> depressive episodes, higher intake of ready-packaged bread and processed meats -> anxiety disorders.	– Public health recommendations to avoid overall UPF intake – Reinforces dietary guidelines promoting minimally processed foods. – Advocate for clinical counseling and population-level nutritional guidance focused on reducing UPF consumption to prevent both cardiometabolic and mental health disorders	(104)
–	– Systemic inflammation – Disruption of gut microbiota – Dopamine dysfunction – Insulin resistance – Oxidative stress – Toxic advanced glycation end-products	– High-quality reviews found a consistent association between UPF consumption and increased risk of depression and anxiety – One review with meta-analysis of prospective studies found a 22% higher risk of subsequent depression (20)	–	(59)
–	– UPFs displace nutrient-dense foods with saturated/trans-fat, sodium, refined carbs and added sugar – Refined carbs, sugar, hydrogenated fats, and preservatives promote neuroinflammation. – High palatability of UPFs alters dopaminergic pathways, which is associated with depressive symptoms – Additives such as emulsifiers may cause gut microbiota dysbiosis, triggering systemic inflammation	– Higher UPF intake -> higher odds of depression – No significant association with anxiety – Processed foods associated with lower odds of depression and anxiety (attributed to the fact that this included healthy foods such as cheese, canned fish, fruit and vegetables, roasted nuts)	– Reduce UPF consumption through public health strategies. – Promote diets rich in minimally processed and nutrient-dense processed foods. – Encourage further longitudinal research to confirm causality and dose–response relationships.	(90)
–	– Nutritional inadequacy (high in energy, saturated fats, salt, and free sugars, while being lower in several micronutrients and fiber) – UPFs can induce metabolic endotoxemia, increase inflammatory cytokines, and impair endothelial function – Alter neurotransmission by changing the availability and activity of dopamine, serotonin, and glutamate–may alter reward pathways and reduce levels of dopamine and serotonin linked to depression and food addiction – Negatively affect the gut microbiome, potentially leading to chronic inflammation and gastrointestinal disorders (IBD, IBS), which are also linked to mental health – Additives, new contaminants from processing, and chemicals, e.g., bisphenols and phthalates from packaging, may cause DNA damage and have a toxic effect on the nervous and immune systems	– Increasing UPF intake significantly worsens mental health (measured in general using the SF-36 scale) – Dose-dependent effect of UPF consumption	–	(98)
–	– High intake of artificial sweeteners (aspartame, saccharin) and MSG → dysregulate dopamine, norepinephrine, and serotonin → mood disorder risk. – TiO_2_ → ↑ IL-6 in plasma and cortex → neuroinflammation; may damage dopaminergic neurons (rats). – Childhood BPA exposure → endocrine disruption → increased anxiety and depression later in life. – Chronic stress ↔ mood disorders → HPA axis dysregulation → altered appetite hormones (noradrenaline, cortisol) and hypothalamic neuropeptides (CRF) → emotional eating.	– Higher UPF intake -> increased cross-sectional odds of depressive and anxiety symptoms, both when assessed together (combined as CMD) and separately – Also increased risk of subsequent depression (from prospective meta-analysis)	– More prospective research to determine directionality – Use of observational studies carries the risk of residual confounders; however, interventional studies may be difficult to ethically justify – Recommends addressing UPF consumption in new dietary guidelines – Supports Mediterranean and other anti-inflammatory diets	(20)
–	– Nutrient displacement (supported by results that participants with higher UPF intake on average had lower intakes of protein, fiber, fruits and vegetables) – AGEs and artificial additives (carboxymethylcellulose, polysorbate-80, saccharin, sucralose) may contribute to gut and metabolic disease -> linked to mental disorder – MSG, and the artificial sweeteners, aspartame and saccharin, cause dysregulation of the HPA axis and alter dopamine, norepinephrine and serotonin synthesis and release	– Higher UPF intake ->22% increased risk of subsequent psychological distress (measured using K10)	–	(61)
–	Micro-nutrient deficiency that affects brain health, an anti-inflammatory diet, and modulation of the gut-microbiome axis	Increased intake of processed meat aRR per serving and sodium is associated with a higher risk of anxiety. Fruits, veg, nuts, and seeds are protective UPF had no significant RR after adjustment	Processed meat increased depression and anxiety risk	(71)
–	Possibly higher SSB drunk by boys -> more results Individuals who consume more sugary food, to stress and negative emotions –> mental disorders are more likely to drink SSBs	Sugar-sweetened beverages ≥1/day positively associated with depressive symptoms, OR 2.28 (1.3–4.01), even stronger association for males specifically OR 3.42 (1.47–7.92) Social anxiety association is not statistically significant Higher body composition increased depression risk for boys who drank sugar beverages ≥1/day	Suggest labels and education programs against SSB	(65)
–	– Gut dysbiosis → ↓ BDNF synthesis → reduced synaptic plasticity → ↑ depression risk and ↓ responsiveness of glutamate/excitatory neurons. – Chemicals in plastic may further increase depression risk. – Neuroinflammation contributes to these effects.	UPF increased depression risk RR 1.28 (1.19–1.38), anxiety not significant Increase in 10% UPF consumption, 11% higher risk of depression, positive linear association ^***^	UPF vs. depression supported with dose dose-dependent relationship	(22)
–	– High-fat diets → ↑ BBB permeability, ↑ pro-inflammatory cytokines, ↓ antioxidant capacity → ↑ risk of depression and anxiety.	High high-fat diet and fructose worsen lipid profiles in mice	Mechanisms provided for depression, anxiety	(107)
–	– Highly palatable foods, reward from UPF	UPF consumption 1.45 (1.06–1.92) is more likely to experience anxiety UPF consumption 1.31 (1.08–1.60) for depression symptoms	More education for pregnant women	(79)
–	– Lack of anti-inflammatory properties e.g., fiber, polyphenols, omega-3 fatty acids, vitamins and minerals	Salty snacks and sweet snacks most consumed UPF, followed by margarine, soft drinks, and meat products “Feeling sad” and “feeling life is not worth living” are most strongly associated with UPF consumption in boys. Nearly all mental health symptoms were significantly associated in girls		(106)
–	–	UPF (5–7/7) vs. Depression^**^, UPF (5–7/7) vs. Anxiety ^**^	Benefits of healthy, and detriments of UPF, identified	(114)
–	–	MSG in UPF may be an excitatory neurotoxin, and affect nervous system function for depression, anxiety, in animal studies	–	(103)
–	–	No significant association for UPF vs. depression or anxiety, minimally processed foods can be protective	Consumption of healthier foods has reduced the risk	(63)
–	–	– Fast food, fried foods are associated with increased depression risk, but no risk for anxiety – Sweetened drinks can increase the risk of depression and anxiety	–	(67)
–	Proinflammatory markers Highly palatable foods increase food anticipation, and binge eating type behavior and loss of self-control	Servings, energy and weight proportion with dose significantly associated with depression and anxiety (individually)	–	(179)
–	– Shortening and nNOS increase related depression excess NO can cause excess signaling, reactive nitrogen species and cell toxicity	Intervention: shortening (partial hydrogenation of palm oil, widely used in fast food) 9 weeks: reduced short-term spatial working memory, anxiety-like behavior, obesity with cognitive impairment, mood disorder 12 weeks: depression like behavior + elevated nNOS protein expression in hippocampus + prefrontal lobe nNOS inhibition improved depression-like behavior	Depression in obese mice	(97)
–	– UPF have a faster energy intake rate, which can affect satiety	UPF vs. depression and anxiety associated with higher risks	–	(87)
–	– High fructose corn syrup → ↑ metabolism, hypertriglyceridemia, insulin resistance, blood glucose fluctuations, and oxidative stress.	< 60 years old: sugar-sweetened beverage, and artificially sweetened beverage were associated with a higher risk of depression disorder coffee, fruit, veg juice protective nil anxiety associations	Age-specific sugar-sweetened beverage vs. depression supported No significant beverages for anxiety	(68)
–	–	UPF vs. mental psychological disorder found mental illness not specified	UPF vs. mental illness supported	(180)
–	– Gut-brain axis, the gut producing signaling molecules, and neurotransmitters may contribute to depression and anxiety	A pro-inflammatory dietary pattern may increase the risk of depression or anxiety	-	(181)
–	– Processing → novel food matrices and bio-incompatible compounds (e.g., advanced glycation end-products) → neurotoxicity and proinflammation. – HPA axis dysregulation and endocrine imbalance → insulin resistance and leptin dysfunction.	High UPF intake led to increased risk of depression and anxiety disorder (individually) and mental health symptoms	UPF vs. depression, anxiety supported	(96)
–	– Western diet → high-temperature processing → Maillard reaction → AGEs, heterocyclic amines, acrylamide → ↑ ROS and inflammation.	Western diet or red meat or refined grains, or SSBs vs. anxiety–no association Western diet vs. depression and depressive symptoms- significant association Fast foods and depressive symptoms/depression. Significant association between red meat, SSBs vs. depression/depressive symptoms, no association Refined grains vs. depression significant association	Advise against the Western diet pattern for the risk of depression	(182)
–	– Acrylamide – Polycyclic aromatic hydrocarbons (PAHs) neurotoxicity in processed foods	Smoked food (long-term high consumption) vs. depression or 2.01 (1.33–3.03) Fried food (Recent moderate consumption frequency with occasional high frequency in the early stage) vs. anxiety OR 1.91 (1.12–3.25) Food with more additives (long-term high consumption frequency) vs. anxiety and depression was significant	Processed foods vs. depression, anxiety supported	(70)
	– Industrial additives for preservation, flavoring and coloring modify the neuronal mitochondrial function by various metabolic pathways, leading to inflammatory processes and neurotransmitter deficiencies – UPFs are also linked to CNS demyelination	– Higher UPF consumption in adolescents -> higher odds of CMD (anxiety and depressive disorders) – The highest quartile showed 20% greater association	– School environment has a significant impact on health -> need to promote healthy eating habits in school and curb profit-driven marketing	(94)
**Depression and other disorders**				
–	– Artificial sweeteners promote purinergic transmission, which is associated with depression – Gene/environment interaction with the AFC and benzoate can be related to the behavior. AFCs may trigger histamine release, which can affect the CNS, sleep and behavior	UPF linked to depression Artificial food colorants (AFCs) and benzoate preservatives may affect those with conduct disorders e.g., ADHD	AFCs and benzoate preservatives should not be used in children	(100)
– Triglycerides – Phospholipid	– Ether phospholipids [e.g., PC (O-34:3)] act as endogenous antioxidants. Lower levels of these lipids in patients suggest reduced antioxidant defense, leading to more oxidative stress. -> induce inflammation e.g., activation of the inflammasome and release of proinflammatory cytokines -> neuroinflammation – Elevated triglycerides [TG (50:1), TG (16:0/18:0/18:1)] may reflect metabolic comorbidities (e.g., fatty liver, weight gain) common in severe mental illness.	– Significant alterations in plasma lipid profiles in patients (with serious mental disorders) vs. controls, including elevated triglycerides, decreased ether phospholipid -> correlated significantly with inflammatory mediators	–	(23)
–	– UPFs → ↑ body fat and low-grade inflammation due to low antioxidants, anti-inflammatory compounds, prebiotics, fermentable fiber, and poor nutritional density. – Plastic packaging and additives → ↑ inflammatory biomarkers (CRP, IL-6, IL-10). – Microglial activation by LPS, IFN-γ, β-amyloid, α-synuclein → neuroinflammation, ROS, nitric oxide → neurotoxicity. – UPFs affect the amygdala-hippocampal complex → impaired emotion regulation. – Thermal treatment → AGE and ALE → oxidative stress; AGE → BBB disruption and brain immune cell damage. – Acrylamide → oxidative damage, ↑ ROS. – UPFs may induce epigenetic changes: altered DNA methylation, histone acetylation, miRNA modulation → impacts lipid metabolism. – Nutritional deficiency, additives, and epigenetic changes from UPFs may contribute to neurodevelopmental disorders (autism, ADHD).	UPF increased depression and anxiety risk	–	(36)
– Trans fatty acids – Saturated long-chain fatty acids: – Short-chain fatty acids	– Trans fats: distort brain membrane phospholipids → impair neuronal signaling. – Disrupt lipid metabolism → inflammation, endothelial dysfunction, visceral fat, insulin resistance. – In pregnancy/lactation → oxidative stress and neuroinflammation (cortex, hippocampus) in offspring → anxiety, memory issues. – Saturated fats: Activate microglia and astrocytes → neuroinflammation. – SCFAs: UPF-induced gut dysbiosis ↓ SCFA → weakens BBB and impairs serotonin/dopamine synthesis. – Neurotransmission: Inflammation or sucralose-induced dysbiosis shifts tryptophan metabolism → ↓ serotonin, ↑ neurotoxic quinolinic acid → poor stress coping and depression risk. – Bisphenols dysregulate dopamine/serotonin genes; perinatal exposure alters placental–fetal tryptophan metabolism → anxiety and ADHD. – Neuroinflammation/toxicity: Brain inflammation disrupts BBB → nanoparticles (TiO_2_, silver) cross BBB → neuronal/glial damage. – TiO_2_ toxic to hippocampal and substantia nigra neurons → impaired memory, learning, movement. – Silver nanoparticles accumulate → short- and long-term memory deficits. – Eating behavior and neural networks: UPF-related dopamine/serotonin disruption → habitual, compulsive eating. – High-fat/sugar intake ↓ reward response → reinforces habits. – High sweetness/fat ↓ sensory satiety → ↑ UPF preference. – Low/non-caloric sweeteners fail to activate reward circuits → underestimate calories, prolong eating. – UPF marketing cues activate reward/motivation regions (insula, OFC, nucleus accumbens) → override control.	– Higher UPF consumption with increased risk of depression, FA traits in longitudinal studies	– More comprehensive evidence on long-term UPF exposure is required to clarify the impact on brain development and mental health – Special attention should be given to childhood and adolescence (critical neurodevelopmental windows) – Easy accessibility poses a significant problem for individuals with impaired executive function, such as a lack of inhibitory control (e.g., in mental illness)	(24)
–	– Withdrawal → ↓ dopamine in nucleus accumbens. – Animal studies show ↑ corticotropin-releasing factor → heightened stress response.	– Animal studies show that frequent UPF intake downregulates dopamine D2 receptors, promoting tolerance and continued consumption. – Human studies: similar findings to animal. Lower striatal responses from SSB after prolonged consumption – Tolerance to fats and salts, and gastric capacity, was also observed Withdrawal: – Animal studies: strong evidence of UPF-induced withdrawal, e.g., paw tremor, head shaking with indicators of anxiety, depression, enhanced craving, binge eating – Human studies: anecdotal withdrawal symptoms reported	Evidence for UPF as a food addiction	(99)
–	– Many UPFs → nutrient deficiencies, oxidative stress, BBB disruption, endocrine dysregulation, impaired brain integrity, ↓ BDNF, altered serotonin and dopamine transmission → depression risk. – ↓ Leptin from UPF consumption → hedonic eating, dopamine sensitization, altered reward systems. – High UPF availability → cue-induced cravings.	High UPF consumption is associated with depression and anxiety Increasing minimally processed food intake was protective UPF consumption increased caloric intake by 500 kcal/day, and participants gained weight on it UPF may be a precursor to binge eating in children	Consider the role of UPF in eating disorders and binge eating	(88)

^**^Refers to statistical significance with *p* < 0.01. ^***^Refers to statistical significance with *p* < 0.001.

#### 3.2.3 Mechanisms of UPF and depression

##### 3.2.3.1 UPFs and dysregulated lipid metabolism

**Figure 4:**
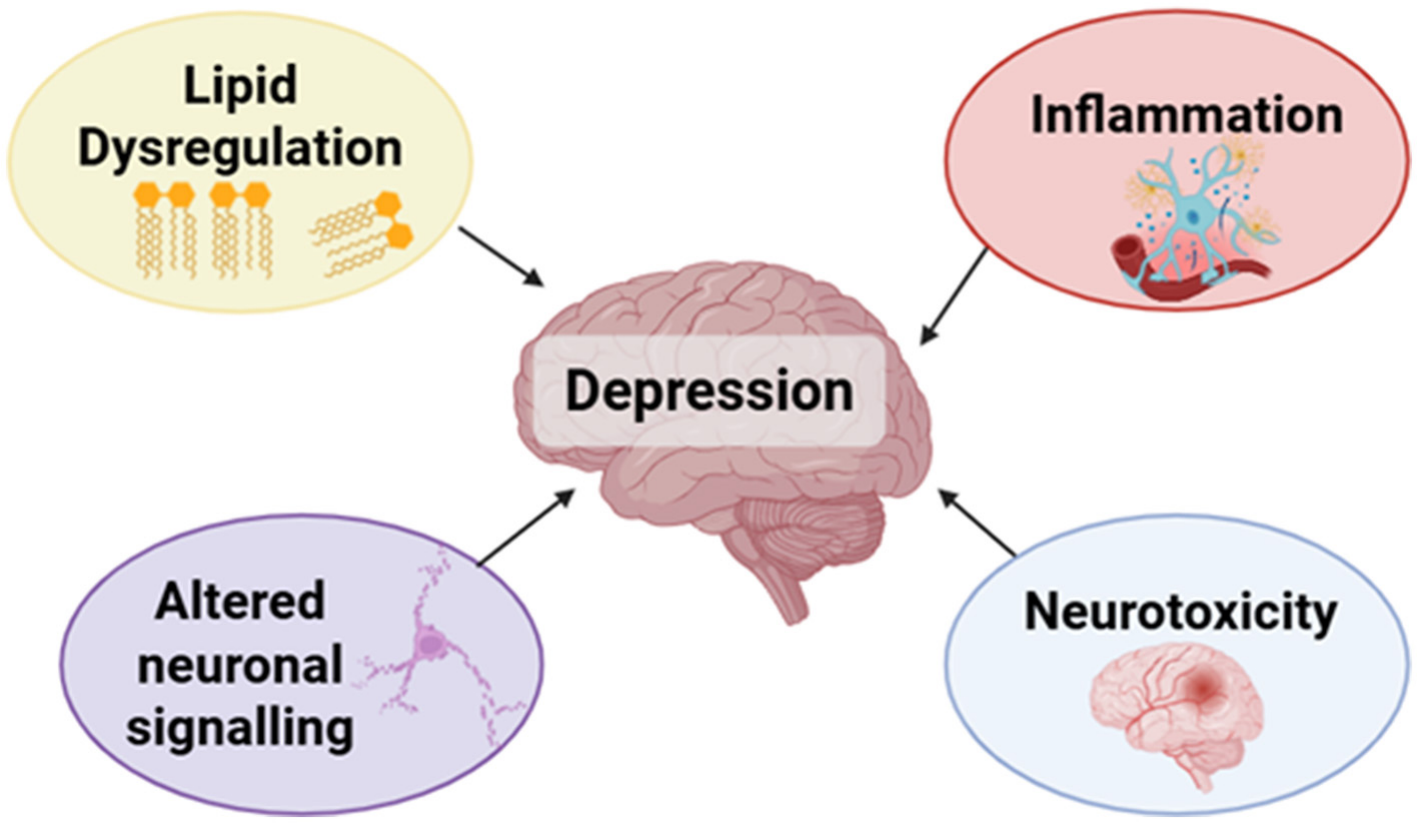
Summary of mechanisms relating to depression induced by UPFs. Mechanisms include lipid dysregulation, inflammation, altered neuronal signaling, and neurotoxicity.

**Figure 5:**
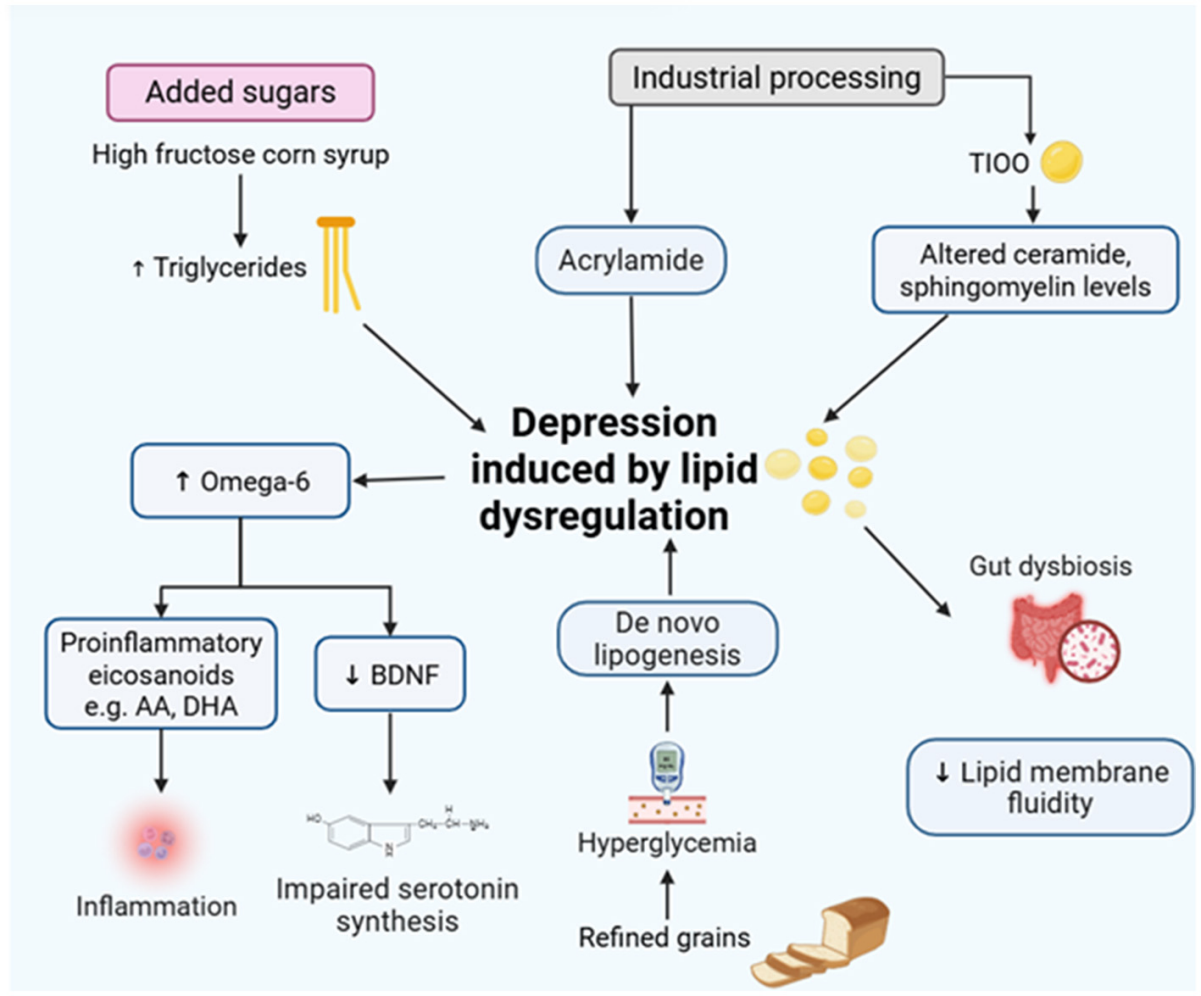
Mechanisms by which UPFs induce lipid dysregulation. UPF constituents that contribute to lipid dysregulation include high-fructose corn syrup, acrylamide and refined grains. Lipid dysregulation leads to inflammation, impaired serotonin synthesis and gut dysbiosis. TIOO, thermo-induced oxidized oil; BDNF, brain-derived neurotrophic factor; AA, arachidonic acid; DHA, docosahexaenoic acid.

Several mechanisms have been proposed to link increased UPF consumption and depression risk (Figure 4; Table 1). A leading hypothesis suggests that UPFs may disrupt normal lipid metabolism, contributing to lipid profile dysregulation that adversely affects brain function and mood regulation (23) (Figure 5). Individuals consuming diets high in UPF content have been shown to exhibit altered lipid profiles, including shifts in key lipid species involved in neuroinflammation, cell membrane integrity, and neurotransmitter signaling, all of which may contribute to depressive symptoms (77). In animal studies, mice fed thermo-induced oxidized oil (TIOO) diets displayed disrupted ceramide and sphingomyelin levels (86). Moreover, the high omega-6 fatty acid content typical of UPFs promotes the formation of proinflammatory eicosanoids, further exacerbating metabolic and inflammatory imbalances (46). High-fructose corn syrup can increase metabolism, hypertriglyceridemia, insulin resistance, blood glucose fluctuations, and oxidative stress (87). Similarly, in another animal study, grain-fed rats exhibited hyperglycemia due to *de novo* lipogenesis, which led to abnormal lipids and contributed to inflammation and impaired neuronal function (52). The worsened lipid profile was reproduced in Lutz, Arancibia (36). Deranged lipid profiles were present in severe mental illnesses but could be influenced by metabolic comorbidities (23). Altered lipid levels may, in turn, trigger systemic inflammation, leading to endothelial dysfunction, increased visceral adiposity, body weight, and insulin resistance (24).

Notably, the consumption of UPF can adversely affect the permeability of important lipid membranes, including the BBB (88). The production of UPF relies on industrial hydrogenation of vegetable oils that form TFAs, which alter the BBB and lead to changes in neuronal communication (24). UPF also result in the reduction of SCFAs that support the BBB (89). The tight junctions of endothelial cells become impaired, facilitating the passage of nanoparticles into the brain and causing damage (24). UPF increases malondialdehyde (MDA) and decreases glutathione (GSH) levels, leading to oxidative stress, lipid peroxidation and reduced lipid membrane fluidity and permeability (52).

##### 3.2.3.2 UPFs and increased inflammation

Refined carbohydrates, sugars, and hydrogenated fat (90) have been identified as contributing to neuroinflammation in the context of excessive UPF consumption (62, 84). UPF consumption over the course of pregnancy and lactation has also been associated with causing neuroinflammation (24). Saturated long-chain fatty acids activate inflammatory signaling of microglia and astrocytes (24). Abnormal microglial function was also identified in mice treated with TIOO (36). Saturated fatty acid-mediated inflammation can alter the neural circuits that control energy balance, leading to excess nutrient consumption and impaired mood regulation (35). Lower levels of brain-derived neurotrophic factor (BDNF) have been identified (43, 46, 89, 91–93). Disrupted BDNF synthesis reduces synaptic plasticity (46), as well as glutamate and other excitatory neuron responses, which may contribute to the pathogenesis of depression (22). UPF have also been linked to the development of demyelination of the CNS (94).

**Figure 6:**
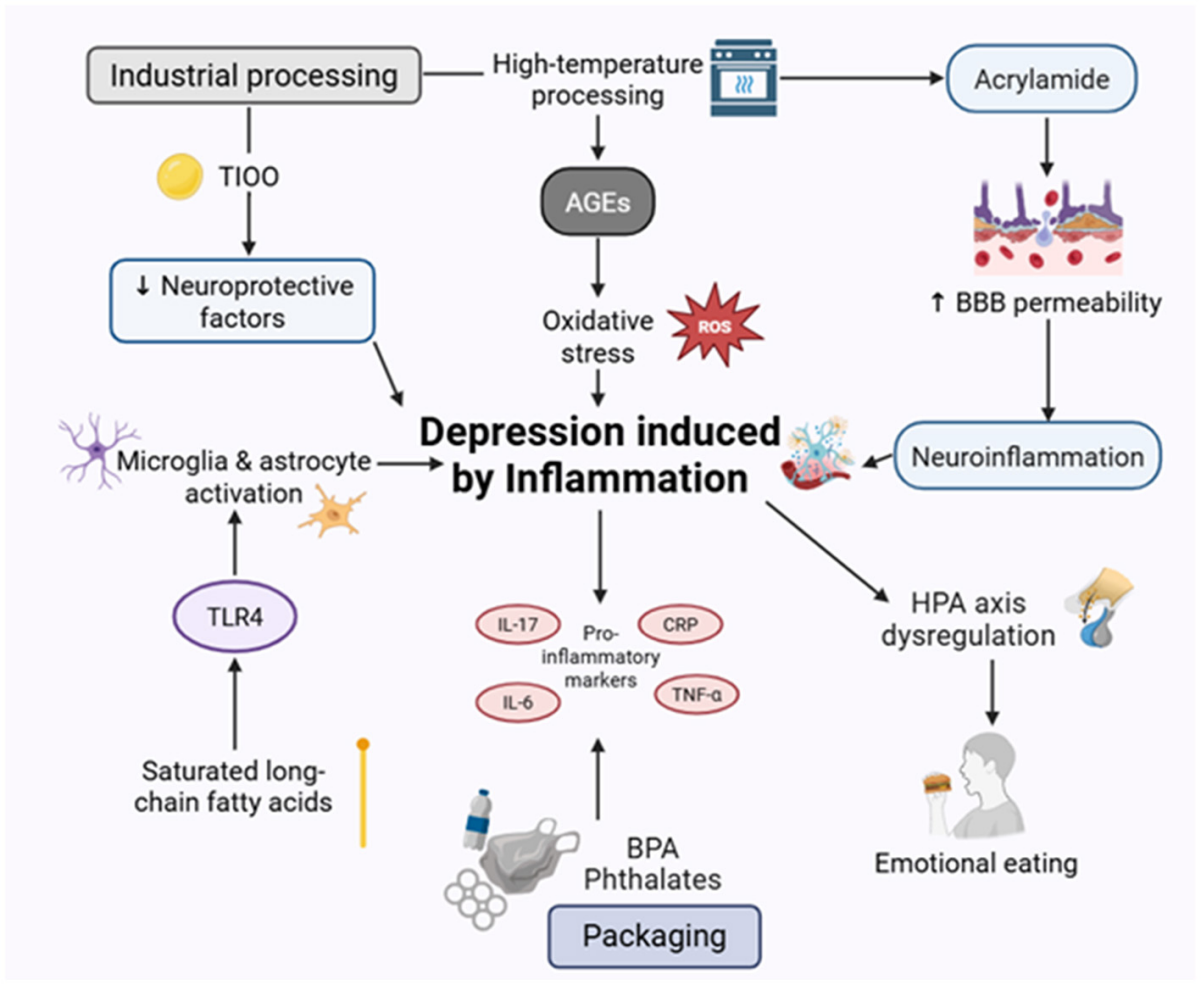
Pathophysiological mechanisms that underpin how UPFs trigger inflammation across different regions of the CNS, thereby contributing to depression. Constituents involved include by-products of industrial processing, contaminants from plastic packaging, and saturated fatty acids. TIOO, thermo-induced oxidized oil; AGE, advanced glycation end-products; ROS, reactive oxygen species; BBB, blood-brain barrier; TLR4, toll-like receptor 4; IL-17, interleukin 17; IL-6, interleukin 6; TNF-α, tumor necrosis factor alpha; CRP, C-reactive protein; BPA, bisphenol A; HPA, hypothalamic-pituitary-adrenal axis.

The association between the consumption of UFP and depressive symptoms has been linked to changes in systemic inflammation (43, 73, 93). Added sugar, sodium, saturated and TFAs have been found to have a number of pro-inflammatory properties (80) (Figure 6). Elevated white cell count was significantly associated with depression symptoms, mediated by inflammation from UPF (30, 95). SFAs act similarly to bacterial lipopolysaccharides (LPS), activating TLR4 on macrophages and microglia (35). They can also cross the BBB and activate TLRs in the brain (35). This can stimulate inflammatory pathways, leading to the phosphorylation of the IkappaB alpha protein and the production of inflammatory cytokines (35). Elevated markers, such as C-reactive protein (CRP), IL-6, IL-17, and TNF-α, have been identified. Microglial activation can lead to neurotoxicity (36). TIOO mice also exhibited downregulation of neuroprotective factors, including Interleukin 4 (IL-4), Interleukin 10 (IL-10), and M2 microglia (86). Eicosapentaenoic acid (EPA), an omega-3 polyunsaturated fatty acid (PUFA), is anti-inflammatory and has an inverse relationship with inflammatory markers and depression (89). The EPA may reduce inflammatory cytokines, such as TNF-α, IL-6, and IL-1β, by inhibiting the NF-κB pathway (43).

AGEs (61) are produced in high-temperature cooking such as frying, roasting, and grilling (known as the Maillard reaction) (8, 61). AGEs are proteins or lipids that glycate and bind to the Receptor for Advanced glycation end-products (RAGE) and mediate inflammatory pathways and reactive oxygen species (ROS) (8). AGEs impair the BDNF-TrkB pathway, thereby impairing neuroplasticity, and cause endothelial dysfunction by forming cross-links in the arterial wall and collagen, leading to stiffness and impaired brain microcirculation (8). AGE can adversely impact BBB integrity and immune cell function (36). AGE-RAGE interaction generates free radicals, pro-inflammatory cytokines, and NF-kB activation. Leading to mitochondrial dysfunction, ROS and apoptosis (8, 96) (Figure 6). Increased RAGE downregulates detoxification pathways and activates glial cells (8). Hyperglycaemia can increase AGE generation (52). But UPF consumption is associated with high levels of AGEs, and AGEs have a strong association with MDD and a higher mortality risk of MDD. Shortening (part of the Maillard reaction) increases neuronal nitric oxide synthase (nNOS) activity, which can lead to excessive signaling, reactive nitrogen species, and cell toxicity (97). The inhibition of nNOS reduced depressive behaviors in animal models (97).

##### 3.2.3.3 UPFs and altered neuronal signaling

**Figure 7:**
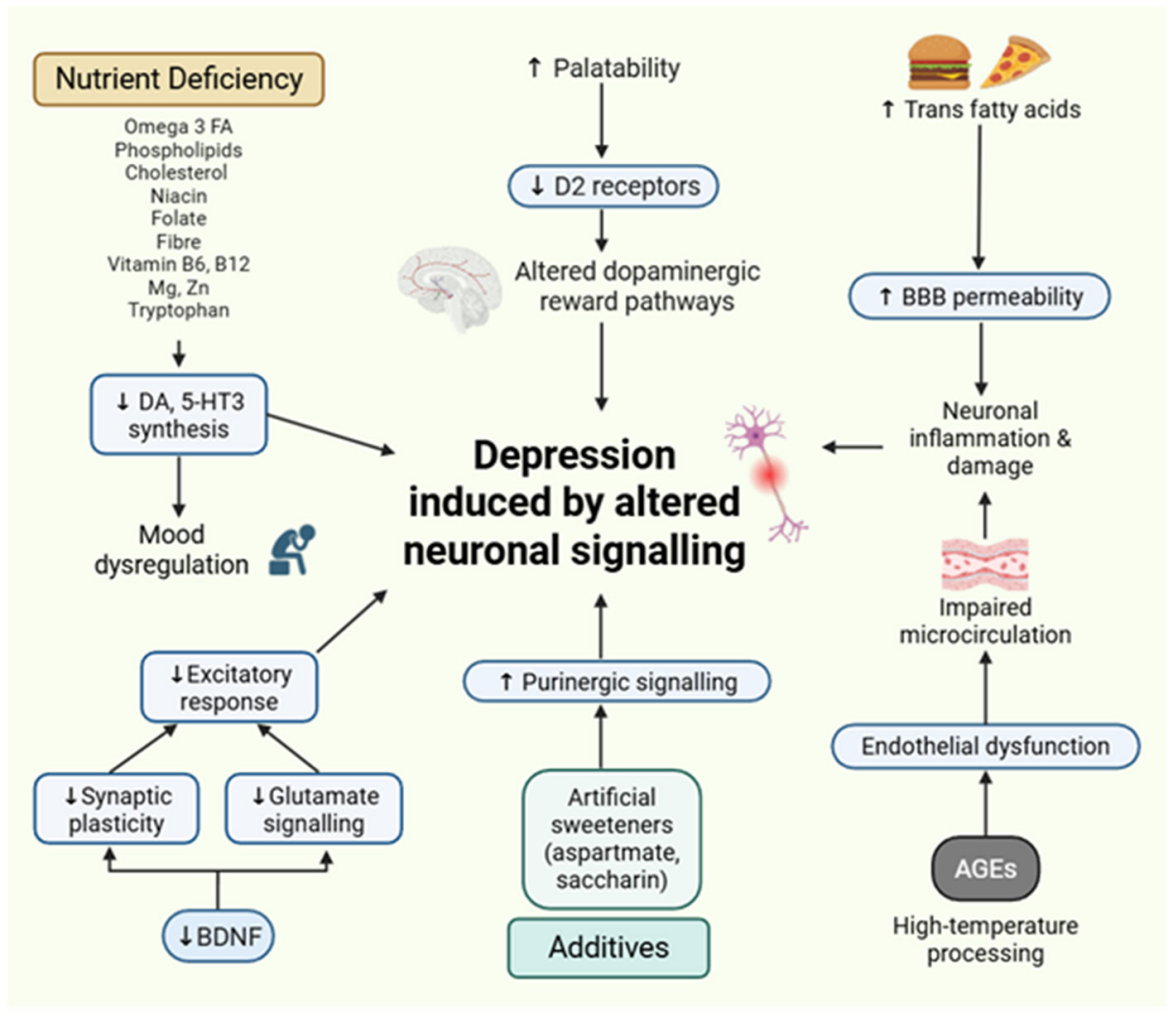
Mechanisms by which UPFs cause depression, via altered neuronal signaling. Altered neurotransmission includes downregulation of dopamine, decreased excitatory response and increased purinergic signaling. UPFs also cause endothelial dysfunction and increased blood-brain barrier permeability, which can lead to neuronal damage. FA, fatty acid; Mg, magnesium; Zn, zinc; BDNF, brain-derived neurotropic factor; D2, dopamine receptor D_2_; BBB, blood-brain barrier; AGEs, advanced glycation end-products.

The consumption of UFP may lead to changes in dopamine and serotonin signaling, which may, in turn, contribute to a heightened risk of mood disorders, including depression (52, 79, 91, 94, 98) (Figure 7). This may occur due to prolonged exposure to inflammatory cytokines (76). Gut dysbiosis and inflammation can alter tryptophan (the precursor to serotonin) metabolism to the kynurenine (KYN) pathway, which is linked to the development of a range of different mental illnesses (24). High palatability may alter dopaminergic pathways (90) or reward pathways (61, 62, 79). Tolerance occurring due to a downregulation of D2 receptors has been identified in human and animal studies, and withdrawal has been apparent in animal studies, e.g., observed tremor, signs of depression, and increased corticotropin-releasing factor (CRF) (99). Other signaling mechanisms may be necessary due to the abnormal dopamine and serotonin signaling. In particular, artificial sweeteners promote purinergic signaling (100), which is associated with depression (66). Mice treated with TIOO have been found to have changes across glutamate signaling pathways (86).

##### 3.2.3.4 UPF additives induced depression

**Figure 8:**
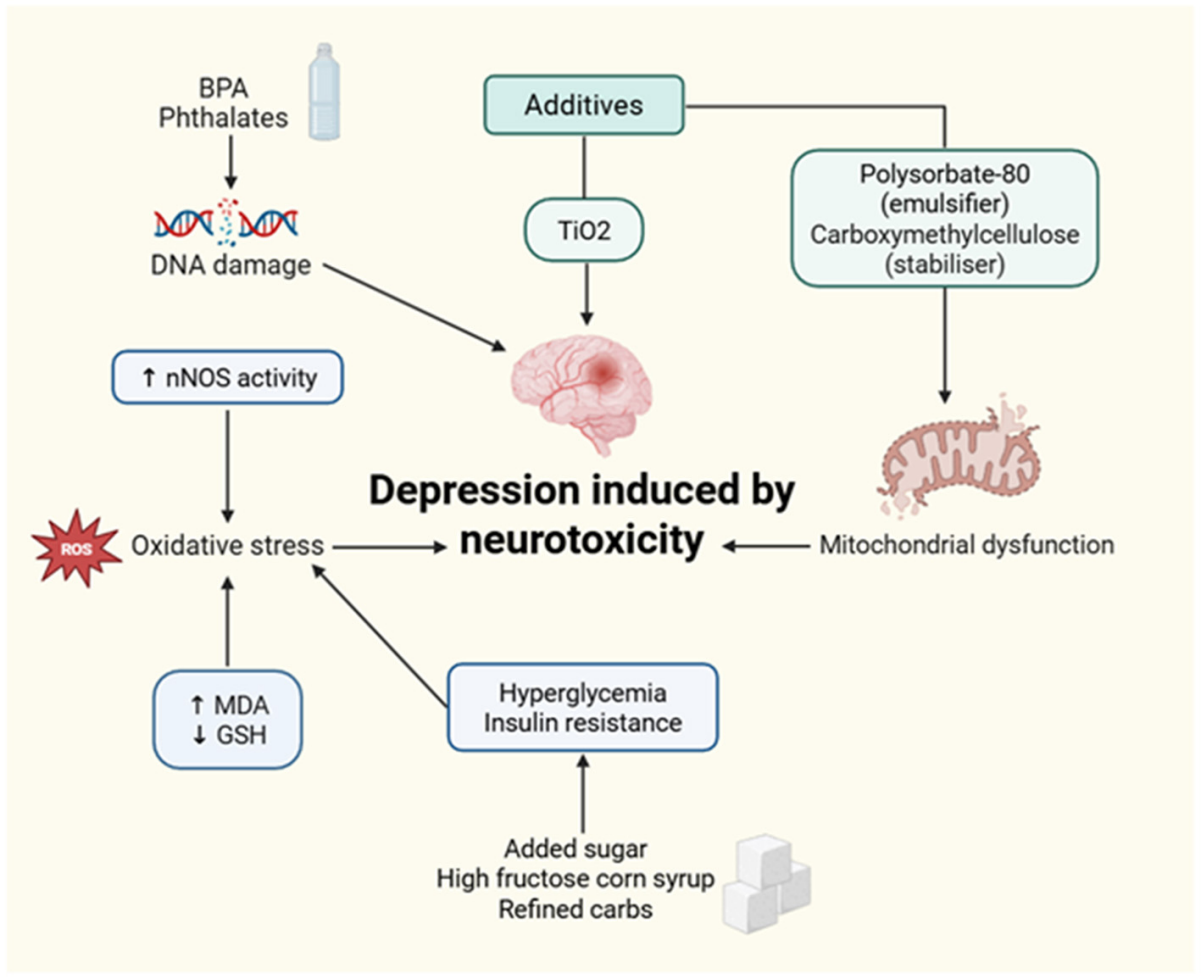
Mechanisms by which UPFs contribute to neurotoxicity-induced depression. Key constituents include plastic components (BPA, phthalates), additives, added sugar and refined carbohydrates. BPA, bisphenol A; DNA, deoxyribonucleic acid; nNOS, neuronal nitric oxide synthase; ROS, reactive oxygen species; MDA, malondialdehyde; GSH, glutathione; TiO_2_, titanium dioxide.

The additives and flavoring compounds which are used in UPF production have been associated with a higher risk of depression (101). Preservatives, flavorings and colourings may, in some cases, alter the mitochondrial function of neurons (94) (Figure 8). These include polysorbate-80 and carboxymethylcellulose (61). Titanium dioxide can be cytotoxic to glial cells, hippocampal cells and dopaminergic substantia nigra neurons (20, 24). Silver can accumulate in the brain and impair short- and long-term memory (24). Aspartame and saccharin (artificial sweeteners) can inhibit neurotransmitter synthesis and signaling (20, 61, 102). Artificial sweeteners can increase the beta-theta brainwave ratio, which is linked to negative emotions (81). MSG in UPFs can be an excitatory neurotoxin that alters the nervous system function (20, 61, 103). Caffeine can cause nervousness and stimulate emotions in the short term, but can also cause long-term emotional decline (69). Artificial food colorants may trigger histamine release, which can affect the CNS, sleep and behavior (100).

##### 3.2.3.5 Neurotoxic contaminants from UPF processing and packaging

Industrial processes in the manufacturing of UPF may be associated with depression (80). Industrial by-products like acrylamide, acrolein, polycyclic aromatic hydrocarbons and furan can be neurotoxic and cause neuroinflammation (36, 70, 74, 80) (Figure 8). Acrylamide causes lipid metabolism disturbance, impairs the BBB and causes neuroinflammation through multiple pathways (74). These pathways include the formation of oxidative damage and ROS (36). In zebrafish, chronic acrylamide exposure altered the cerebral lipid metabolism and immune responses (74). These also impaired BBB function and caused neuroinflammation (74). These mechanisms were linked to depression-like behavior (74). Acrylamide also has the potential to affect Peroxisome Proliferator-Activated Receptors (PPAR), and Janus Kinase/Signal Transducer and Activator of Transcription (JAK-STAT) signaling, and hence is a chemical with a strong ability to negatively affect the brain (74). Chemicals in plastic packaging may increase the risk of depression (22). Plastic food packaging and additives increase inflammatory biomarkers CRP, IL-6, and IL-10 (36). Contaminants from processing, such as bisphenols and phthalates from packaging, can cause DNA damage and toxicity to the immune and nervous systems (75, 98). They can also interfere with cell signaling and gut barrier function, which can increase permeability and cause inflammation and metabolic changes (104). Exposure to BPA in childhood can be associated with depression later in life through reduction of volumes in the mesocorticolimbic regions (amygdala, cingulate cortex, ventral putamen) (20, 30).

##### 3.2.3.6 UPFs and dietary deficiencies

Dietary deficiencies arising from consuming UPFs with poor nutritional quality (36, 61, 71, 90, 91, 98, 105, 106) and a lack of omega-3 fatty acids, phospholipids, cholesterol, niacin, folate, fiber, vitamin B6, and B12 (76) has been linked to depression. This is because the healthy nutrients are no longer present to support mental health (76). Antioxidants, fiber, and micronutrients, which are missing in an UPF diet, improve inflammation and reduce oxidative stress, which protects against depression (71, 82, 107). Low magnesium and zinc may impair neuroplasticity (43), and low tryptophan (the precursor of serotonin) availability, which may reduce brain serotonin synthesis (46, 76). This can increase depressive symptoms according to the monoamine hypothesis (51, 52). Increased tryptophan metabolism can lead to a shift toward the KYN pathway (24). These lead to an increase in quinolonic acids (neurotoxic), and a decrease in the kynurenic acids (neuroprotective), overall leading to mental illness such as depression (24). The UPF additives can disrupt the gut microbial environment (76). Gut dysbiosis (90, 93, 98), for instance, through the reduction of SCFAs, may affect serotonin and dopamine synthesis (24). Gut dysbiosis through the absence of important micronutrients and antioxidants can contribute to inflammation and altered transmission (71, 80, 93). The UPF saturated fatty acids increase the ratio of gram-negative bacteria, which increases intestinal permeability and thereby inflammation systemically (35, 52).

##### 3.2.3.7 Other proposed mechanisms

Changes across several key brain regions in response to UPF consumption may lead to reduced volumes of the mesocorticolimbic regions (30, 36), left ventral putamen, amygdala, and the dorsal frontal cortex (95). High sugar and low BDNF have been linked to hippocampal atrophy (92). These brain changes can cause impaired ability to gauge the nutritional content of UPF, causing overeating and metabolic dysfunction (78).

Cardiometabolic dysregulation and metabolic syndromes are known to be important risk factors for a range of different mental illnesses (98, 108). Excessive feeding can cause metabolic dysfunction (109), as well as altered peptide signaling for appetite and satiety, including YY (101), GLP-1, GIP, and CCK (109). UPF interaction with the HPA axis has also been proposed (20, 89, 91, 96). Systemic inflammation may lead to activation of the HPA axis, leading to an increase in depression symptoms (105). SSBs with high fructose can cause corticosterone levels to rise, leading to HPA axis dysregulation (110) (Figure 6). Worsened glycaemic indices (109) of UPF causes blood glucose level (BGL) peaks, leading to compensation by cortisol and adrenaline, which can cause a lower mood (84). UPF have faster energy intake rates, which affect satiety and BGL spikes (87).

Some of the proposed pathophysiological mechanisms that have been proposed to account for the relationship between UPF, as well as the risk of mood disorders such as depression, include genetically based factors, including the MC4R gene, CRY1 gene, and Cav1 gene, which are associated with an elevated risk of developing depression (60). However, epigenetic changes, including high fat and sugar content, result in changes in acetylation patterns (36), may have a synergistic effect (60, 100). Oxidative stress can cause DNA damage and telomere shortening (95).

### 3.3 UPF and anxiety

#### 3.3.1 Background on known mechanisms of anxiety

As of 2021, an estimated 4.4% of the global population experiences an anxiety disorder, making it the most prevalent mental disorder (12). Current hypotheses regarding the pathophysiology of anxiety disorders emphasize an imbalance between neuronal excitation and inhibition, characterized by reduced Gamma-aminobutyric acid (GABA) inhibition and excessive glutamatergic activity, which contributes to neuronal hyperexcitability and the manifestation of anxious behaviors (111). Additionally, dysregulation of key neurotransmitters, such as elevated noradrenaline and reduced serotonin, particularly within the amygdala, has been implicated in heightened anxiety and impaired stress resilience (112, 113). High intake of UPFs may contribute to the development of anxiety via both pathways.

#### 3.3.2 Association between UPF consumption and anxiety risk

Findings from previous studies, which have specifically investigated the relationship between UPF consumption and the risk of anxiety disorders, have been mixed, but increasingly, there are several recent findings that have shown a possible association between UPF consumption and the risk of anxiety disorders (Table 1). Several studies report that higher UPF intake is linked to elevated cross-sectional odds of anxiety, either as an independent outcome (79, 114) or within broader measures of common mental disorders (CMDs) that encompass anxiety and depressive symptoms (94, 98, 115). The associations appear particularly robust among adolescents (94) and in analyses demonstrating significant dose-response relationships (98), with specific UPF subgroups such as artificially sweetened beverages, processed meats, ready-packaged bread, high-sodium foods, foods high in additives and fried foods emerging as significant dietary contributors (67, 70, 71, 104, 115). However, the evidence is not entirely consistent. While some studies suggest that UPF consumption is linked to an increased risk of depression, they report little to no association with anxiety, highlighting significant heterogeneity in the findings (65, 67, 90), while others report no significant associations with either outcome (63). Overall, although the findings from the recent literature indicate a plausible connection between UPF consumption and anxiety, the evidence remains inconclusive, particularly in view of the limited number of prospective studies evaluating clinically diagnosed anxiety as a distinct outcome.

#### 3.3.3 UPF and dysregulated lipid metabolism in anxiety

**Figure 9:**
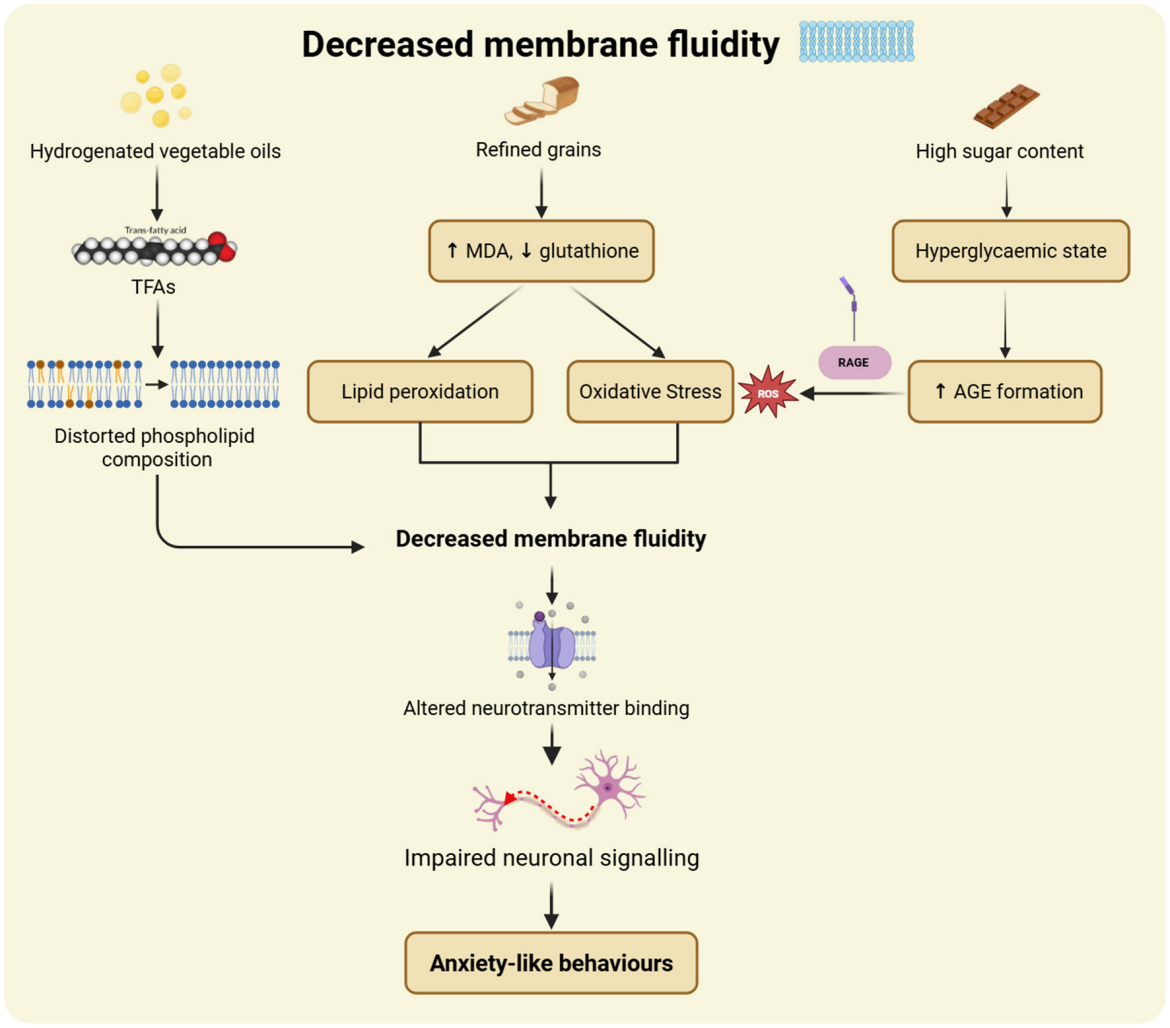
UPFs cause decreased membrane fluidity, leading to impaired neuronal signaling and anxiety disorders. Key constituents involved include hydrogenated vegetable oils, refined grains and high sugar content. TFAs, trans fatty acids; MDA, malondialdehyde; ROS, reactive oxygen species; AGE, advanced glycation end product; RAGE, receptor for advanced glycation end products.

Lipid-related mechanisms may play a significant role in the association between UFPs and anxiety (Table 1). One potential pathway involves altered lipid membrane permeability, which several distinct UPF constituents may mediate. TFAs formed during the industrial hydrogenation of vegetable oils are believed to distort phospholipid composition of the brain membrane, increasing membrane rigidity and hence affecting neuronal signaling (24). In animal models, maternal consumption of trans fats during pregnancy and lactation has been linked to increased neuroinflammation and oxidative stress in the offspring's brain, resulting in anxiety-like behaviors and memory impairments (24). Similarly, high intake of refined grains in rats has been shown to elevate MDA levels and reduce GSH, thereby increasing oxidative stress and promoting lipid peroxidation. These changes reduce lipid membrane fluidity, with increased rigidity impairing neurotransmitter binding, similar to the effects of TFAs (52) (Figure 9). Hyperglycaemia further contributes to membrane rigidity through the generation of AGEs, which aggravate oxidative stress and promote free radical formation, causing damage to the lipid bilayer (52).

**Figure 10:**
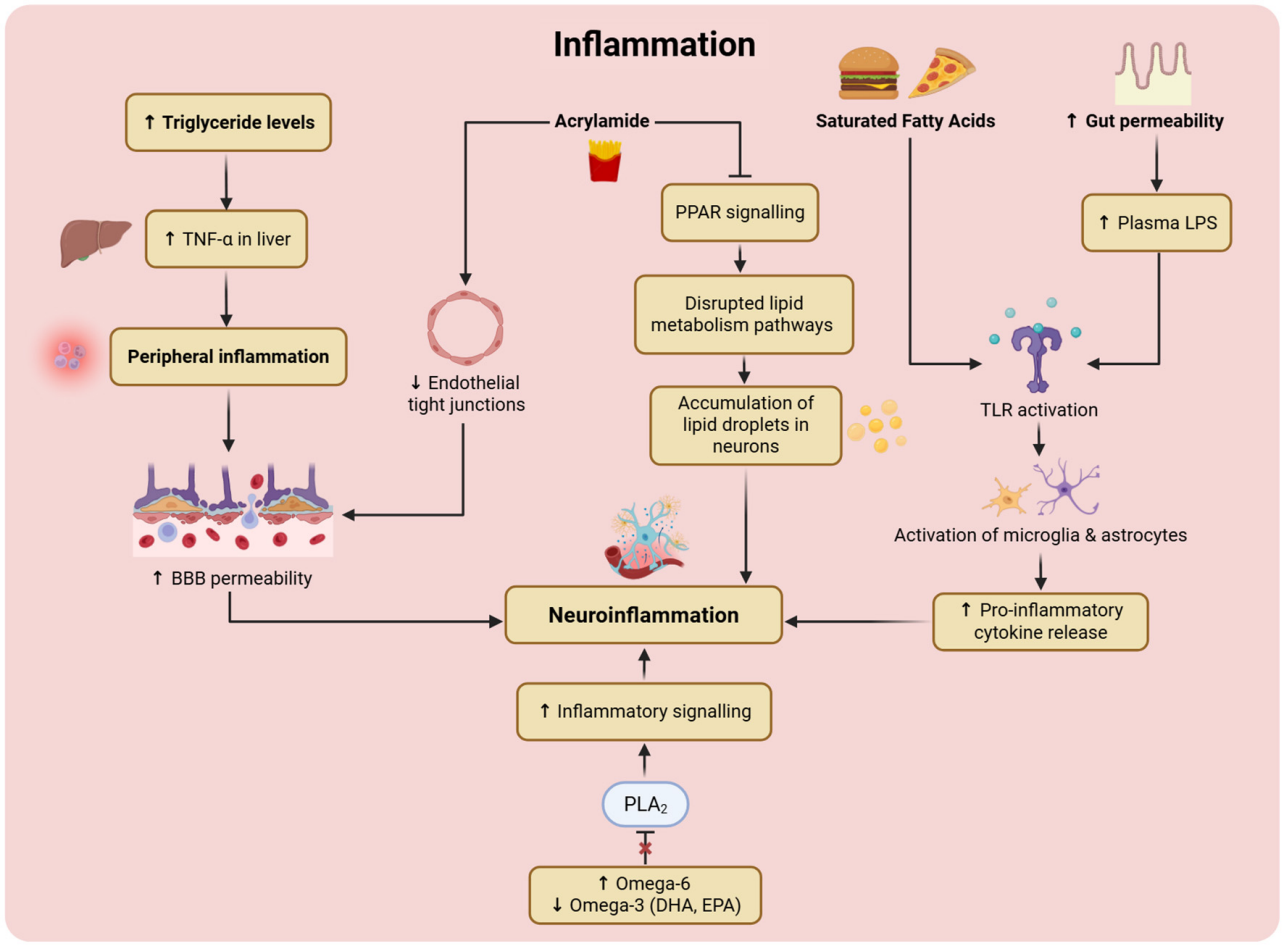
Mechanisms underlying UPF's contribution to neuroinflammation. TNF-α, tumor necrosis factor alpha; BBB, blood-brain barrier; PPAR, peroxisome proliferator-activated receptors; PLA_2_, phospholipase A2; DHA, docosahexaenoic acid; EPA, eicosapentaenoic acid; LPS, lipopolysaccharide; TLR, toll-like receptor.

A second potential pathway involves the interplay between lipid dysregulation and inflammation. Diets high in UPFs may trigger both peripheral and central inflammatory responses, resulting in elevated triglyceride and TNF-α levels in the liver, as well as increased IL-6 concentrations in the prefrontal cortex, as observed in animal models (116, 117) (Figure 10). Peripheral and central inflammation may be linked through the ability of systemic inflammatory mediators to travel through a “leaky” BBB, as UPFs have previously been shown to compromise BBB integrity (117). These inflammatory changes correlated with anxiety-like behaviors in animal models (116, 117). Saturated fatty acids, in particular, were shown to increase cytokine production mediated through the activation of microglia and astrocytes (24). Furthermore, other inflammatory processes, such as metabolic endotoxemia (characterized by elevated plasma LPS) driven by increased gut permeability, may contribute to peripheral and neuroinflammation, manifesting as anxious behaviors (116). The nutrient displacement theory suggests that the displacement of protective anti-inflammatory lipids, such as omega-3 PUFAs (docosahexaenoic acid, EPA), contributes to increased inflammatory signaling via the disinhibition of phospholipase A2, which normally mediates the production of pro-inflammatory mediators (117). Docosahexaenoic acid (DHA) and EPA were shown to reverse elevated TNF-α levels and ameliorate anxious behaviors (117).

The integrity of the BBB may, in some clinical situations, be a lipid-sensitive target of UPFs (Figure 10). Acrylamide, a Maillard reaction contaminant abundant in fried foods, down-regulates endothelial tight-junction proteins, weakening BBB integrity and permitting infiltration of inflammatory mediators, leading to neuroinflammation (74). Beyond altering the permeability of the BBB, acrylamide disrupts multiple lipid metabolic pathways, including cholesterol, arachidonic acid, sphingolipid, and phospholipid metabolism, mainly through the dysregulation of upstream PPAR signaling, which is heavily involved in lipid metabolism (74). These disturbances result in the accumulation of lipid droplets in brain cells, evidencing impaired cholesterol metabolism. Perturbed sphingolipid and phospholipid metabolism led to the upregulation of apoptogenic ceramides and the downregulation of lipids involved in vital cerebral functions, such as neurotransmission, anti-inflammation, and synaptic refinement. Changes in arachidonic acid metabolism appeared to favor an increase in lipid peroxidation, leading to the formation of downstream inflammatory mediators and inducing oxidative stress. This may contribute to anxiety-like behaviors (74).

**Figure 11:**
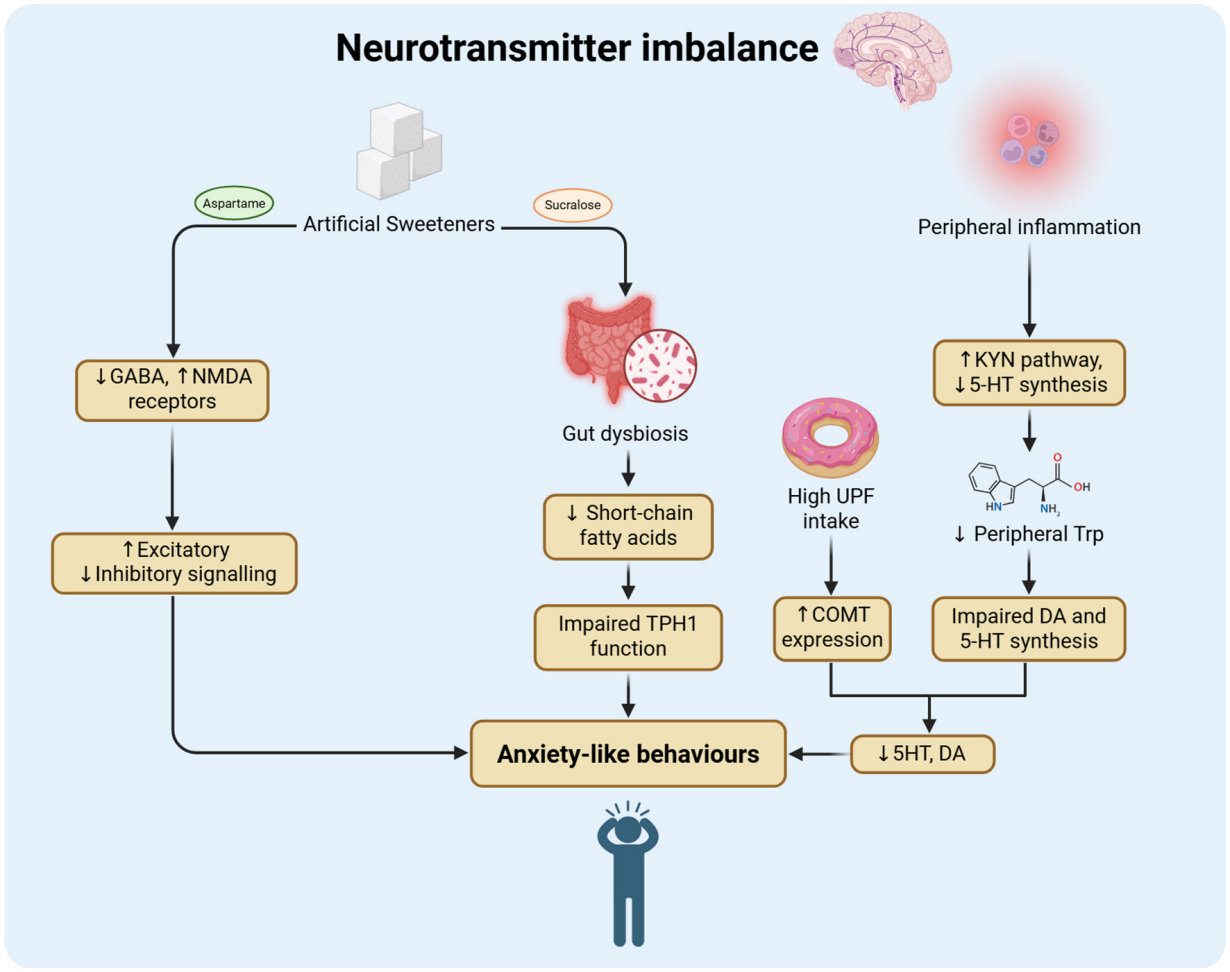
UPFs lead to neurotransmitter imbalance, manifesting as anxiety-like behaviors. Artificial sweeteners and peripheral inflammation play key roles. GABA, gamma-aminobutyric acid; NMDA, N-methyl-D-aspartate; TPH1, tryptophan hydroxylase 1; COMT, catechol-O-methyltransferase; KYN, kynurenine; DA, dopamine; 5-HT, serotonin.

Lipid dysregulation can also impact neurotransmission. Normally, SCFAs produced by beneficial gut microbiota regulate enzymes critical for the synthesis of serotonin and dopamine. However, UPF-induced gut dysbiosis, such as that observed following sucralose exposure, reduces SCFA availability, thereby impairing the regulation of these key neurotransmitters (24). Peripheral low-grade inflammation exacerbates this effect by diverting tryptophan metabolism (usually a precursor for serotonin) toward the KYN pathway, resulting in reduced peripheral tryptophan availability and lower central serotonin synthesis (24) (Figure 11). Endocrine disruptors, such as bisphenols, exacerbate this dysregulation during fetal development by altering placental tryptophan metabolism, with long-term neurodevelopmental consequences for offspring (24). Supporting this, animal studies show that high-UPF diets reduce serotonin and dopamine concentrations, while increasing Catechol-O-methyltransferase (COMT) expression, which accelerates dopamine degradation and produces anxiety-like behaviors (116). In addition, food additives such as aspartame may further impair neurotransmission through altered expression of genes regulating glutamate/GABA signaling in the amygdala (111). This results in excessive excitatory drive, characterized by downregulation of GABA signaling and upregulation of postsynaptic N-methyl-D-aspartate (NMDA) receptors, which has been shown to manifest as anxiety-like behaviors that persist across generations (111) (Figure 11).

Taken together, these findings highlight how lipid dysregulation may be a unifying mechanism linking high UPF intake to pathophysiological changes underpinning anxiety. Disruption of lipid metabolism not only alters membrane integrity and lipid homeostasis but also promotes inflammation, compromises the BBB, and perturbs neurotransmitter systems (Table 1).

### 3.4 UPF and eating disorders

#### 3.4.1 Background on known mechanisms of eating disorders

UPF have been implicated in the development and maintenance of eating disorders (ED), including binge-eating disorder (BED) (118). UPF constitute 60%−80% of the dietary makeup of the diet in ED diets, as well as almost 100% of the food that is consumed in BED (118, 119). UPF consists of a small range of ingredients that enable restrictive eating habits (120). This allows the perseverance of unhealthy habits and adherence to uniformly processed foods (120). The presence of a food addiction (FA) as a comorbid problem in the setting of an ED is generally more severe than when it occurs in the absence of a comorbid ED (121). This may in part be due to a so-called “addiction transfer,” which may occur as a potential coping mechanism in mental illness (91). In fact, FA and BED may, in some cases, co-occur (121, 122). BED, bulimia nervosa (BN) and anorexia binge-purge type have also been found to be associated with a condition known as Loss of Control Binge Eating (LCBE), which has a complex etiology (47).

#### 3.4.2 Effect of UPF on the increased risk of eating disorders

In a previous study, it was found that there was a significant correlation between UPFs and the development of ED (91, 123, 124) in a dose-dependent manner (123) (Table 2). That is, UPF were associated with increased severity of eating disorder (121). Other studies have shown that the consumption of UPF was associated with the development of BN and BED (122, 124) but not in the case of anorexia nervosa (AN) (122). Furthermore, the consumption of UPF was found to be a precursor to ED among children (88).

**Table 2:** Summary of studies implicating increased UPF consumption and dysregulated lipid metabolism associated with autism, ADHD, eating disorders and food addiction.

**Lipid subtypes implicated**	**Mechanisms**	**Key findings**	**Therapeutic implications**	**Source**
**Autism**				
Lipoic acid	– Neuroinflammation, microglia overactivation and synaptic dysfunction are linked to autism – LA suppresses inflammatory responses and possibly gut microbiota, and increases SCFAs and serum amino acids	Acrylamide (ACR) treated rats: elevated neurotoxicity (reversible by alpha-lipoic acid (LA) admin) LA improved ACR-induced cognitive impairment and autism like behaviour LA restored synaptic plasticity in ACR mice neurons, inhibited microglial activation and inflammation LA can regulate gut microbiota and gut barrier function	ACR is a mediator of neuroinflammation and damage in the brain LA may be a suitable alleviator of ACR effects	(160)
–	–	Western diet in pregnancy is not associated with autism	–	(162)
**ADHD**				
– Saturated fats – Omega-3 fatty acids–DHA, EPA – AA (arachidonic acid)	– UPFs high in saturated fat and low in omega-3 → omega-3 (DHA, EPA, n-3 PUFAs, AA) protective in ADHD; supplementation improves symptoms. – UPFs → gut dysbiosis → neuroinflammation → disrupted gut-brain axis → impacts neurodevelopment and behavior. – High sugar → ↓ dopaminergic responses → altered mesolimbic dopamine signaling and cortical inhibition → ADHD symptoms. – Early UPF exposure (age 3–4) → sensitive period for neurodevelopmental impact.	– Increased UPF intake (increase in grams, kilocalories or percentage energy intake) at 3–4 years of age -> predicted hyperactivity/inattention symptoms, but not at 7–8 years	– Implement public policies aimed at reducing the consumption of UPFs across all age groups, especially early childhood	(148)
–	– Prenatal programming: the idea that nutritional exposures during critical fetal development stages can have lasting consequences, through epigenetic modifications	– Maternal consumption of UPF associated with a minor increase (3%) in ADHD symptom score upon follow-up at age 8, and no significant change in diagnosis risk. – However inverse relationship was robust, higher diet quality -> lower ADHD symptom score and risk of ADHD diagnosis	– Improving maternal diet quality during pregnancy as a public health strategy	(147)
–	– Sugary beverages may affect brain development at certain stages in utero, and changes in the dopaminergic system	Sweetened carbonated beverage consumption daily in pregnancy has a weak increase in ADHD symptom score	–	(149)
–	–	High sugar, carbs, and nutrient deficiencies are related to increased ADHD symptoms	–	(183)
–	– Sugar → ↑ blood glucose → short-term energy boost → increased excitement and impulsiveness. – Sugar-rich foods → activate reward system; ADHD is linked to reward system dysfunction.	Processed food-sweets (fried food, processed meat, sugared beverages, candy) were positively associated with ADHD OR: 1.451 (1.041–2.085). Tertile 3 had a higher OR 2.646 (1.213–5.933) The desire to drink and eating behavior positively correlated with ADHD	Processed food vs. ADHD supported	(150)
**ADHD and autism**				
–	– HPA axis dysregulation	UPF vs. ADHD: 1.06^**^(1.03–1.09) UPF vs. Autism 1.10^**^ (1.06–1.14)	–	(151)
		Referred to Kvalvik 2022 for SSB and ADHD		(184)
**Eating disorder**				
–	– UPFs → altered reward processing/signaling in orbitofrontal cortex and inferior frontal gyrus. – High salt, saturated fat, sugar → altered gustatory systems. – UPFs → impaired emotional regulation. – Low/no-calorie sweeteners → ↓ satiation → ↑ overeating. – Modified texture → faster eating → ↑ overconsumption. – Additive-rich UPFs → neuroinflammation → altered dopamine and serotonin transmission → cytotoxicity (in mice).	UPF is associated with binge-eating disorders (BED); those with BED consume a larger proportion of UPF	Mechanisms for UPF relation to binge eating	(124)
– Omega-6 polyunsaturated fatty acids – Trans fats	– Insulin resistance and hyperinsulinemia → ↑ hunger, cravings, fat accumulation. – High omega-6/omega-3 ratio → inflammation, microbiome toxicity → linked to depression and ED. – Additives: propionate → insulin resistance/hyperinsulinemia; transglutaminase emulsifier → intestinal damage and inflammation. – Artificial sweeteners → glucose intolerance and disrupted satiety signaling. – Sugar–fat combinations → supra-additive activation of reward pathways; artificial sweeteners impair calorie estimation. – UPFs ↑ ghrelin → disrupt satiety.	– Over the last 50 years, advanced food science techniques –> invention of cheap, palatable and stable products -> high content of additives such as high-fructose corn syrup -> contributing to the epidemic of NCDs – UPF is strongly linked with the development and maintenance of EDs, forming 60%−80% of patients' diets, and almost 100% of binge eating foods	– Integration of dietary interventions is needed as part of standard ED treatment – Low-carbohydrate, high-fat diets show promise for improving satiety, reducing cravings, stabilizing glucose, and helping BED/BN – Use of continuous glucose monitoring (CGM) for personalized diet planning	(118)
–	– Ghrelin, fasting glucose and insulin levels increased whilst peptide tyrosine hormone decreased on UPF diet – UPF alters neurobiological reward pathways in eating behaviors. Normally, the striatum estimates the calorie content of food, sweeteners/sugars/fats, UPFs disrupt the brain's ability to accurately predict energy intake –> overconsumption	– UPF made up a significant portion of the diet for individuals with eating disorders and caused significant overconsumption when compared to a minimally processed diet – AN associated with insulin sensitivity, BN and BED with insulin resistance – Binge-eating episodes are significantly associated with UPF (100% of episodes involved UPFs)	– Personalized nutritional approach for patients with eating disorders, adapted to individual metabolic responses – CGM can show patients immediate feedback from food intake, help facilitate rapid behavioral change, and be used for remote monitoring.	(119)
–	– High sugar/fat/salt → ↑ palatability → enhanced reward signaling → bulimic and binge-eating tendencies. – Overlaps with FA → impaired control and activation of dopamine reward pathways. – Low fiber → faster eating, ↑ intake, ↓ satiety. – Artificial sweeteners may further promote overeating	– Increased UPF intake -> increased risk of bulimic and BED, but no effect on AN	– Adoption of dietary guidelines to limit UPF consumption – Improve availability and affordability of whole and minimally processed foods	(122)
–	Brain reward response to highly palatable food e.g., through flavor enhancers sugar may affect dopamine and serotonin release, cause mood alteration and pleasure	Sweet foods associated with LOCE and overeating Salty/fried food and pizza/fast food with higher LOCE and overeating Sweetened beverages was linked to higher overeating	Salty, fried foods are associated with loss of control and overeating	(125)
	– UPF are engineered to have higher palatability with high content of calories, fat, sugar, and salt -> may lead to overeating and compulsive food consumption	– Salty UPF in the last 7 days is significantly associated with laxative misuse/self induced in girls – Sweets in the last 7 days are significantly associated with laxative misuse/self-induced vomiting in males and females	– Suggest training school teachers to recognize early signs of EDs–educational campaigns that promote healthy eating habits – Mandatory education about EDs and risky behaviors	(127)
	– UPF have a narrow ingredient range, which allows those with restrictive food intake disorder to adhere to uniform processed foods, allowing them to persevere	PUFA, nutrients in utero, are necessary to support synaptic transmission Maternal consumption leading to fetal BBB compromised by UPF activity Western diet could be related to a smaller hippocampal volume	–	(120)
**Food addiction**				
– Saturated fats – Trans fats	– Human brains evolved to crave calorie-dense foods → sugar and fat activate reward/motivation systems (opioid and mesolimbic dopamine pathways); hippocampus encodes high-calorie experiences more strongly. – Refined carbs → glycaemic spikes and dips → insulin release and hypoglycaemia → ↑ striatal reward activation → promotes overconsumption; prolonged sugar intake ↓ dopamine responsivity (animal studies). – High fat intake → stimulates μ-opioid receptors in ventral striatum → stronger reward responses; saturated/trans fats in UPFs produce greater reward than natural fats. – Carbs + fat → supra-additive reward response; carbs drive dopaminergic addiction, fats enhance palatability and blunt sugar's negative effects (glycaemic spikes, withdrawal).	– Validates the concept of “food addiction” as a substance-based addiction – Individuals meeting FAcriteria show greater depression, impulsivity, and emotion dysregulation – Neuroimaging shows similar patterns of reward dysfunction and inhibitory control deficits between food addiction and substance-use disorders; even after adjusting for BMI, women with higher YFAS scores show greater reward-related activity (caudate, anterior cingulate cortex) in response to highly palatable food cues.	Therapeutic recommendations: – Individualized harm-reduction strategies that identify high-risk foods and contexts (e.g., eating sweets alone in an emotional state) and reducing exposure to such triggers Policy implications: – Tax UPFs and sugar-sweetened beverages – Restrict marketing, especially to children – Limit accessibility, such as zoning laws, to reduce the density of fast food stores near schools – Address the abundance of UPFs in under-resourced communities with limited access to fresh, minimally processed foods	(137)
– Saturated fat – Trans fat (both correlated with FA)	– High fat concentrations initially increase dopamine levels in reward circuits -> promotes UPF-seeking behavior. However, chronic high lipid concentrations -> reduction in dopamine release in the nucleus accumbens -> tolerance – High sucrose also decreased the density of dopamine receptors in reward regions -> also contributing to the development of tolerance	– Children and adolescents with FA consumed significantly higher amounts of UPFs, leading to higher overall energy intake	– Emphasized the need for interventions early in life and changing the obesogenic environment	(138)
– FFAs – Saturated fats	– Overeating → obesity; food addiction and obesity often co-exist. – Obesity → lipoinflammation: WAT expansion → ↑ pro-inflammatory cytokines (MCP-1, TNF-α, IL-6, IL-1β) and M1 macrophage recruitment. – WAT releases FFAs → lipotoxicity → ectopic lipid accumulation (ceramides, diacylglycerols). – Cytokines cross BBB → neuroinflammation in mesolimbic reward circuits. – Saturated fats activate TLR4 → inflammatory pathways. – Inflammation → ↓ striatal dopamine → blunted reward sensitivity → ↑ food “wanting”; ↓μ-opioid receptor → ↓ “liking”/pleasure. – Neuroinflammation → ECM degradation → disrupted synaptic plasticity, dendritic spines, myelination → altered reward systems. – High-fat diet → ↓ occludin-1 → BBB integrity loss; ↑ plasma cytokines correlate with ↑ CSF albumin → BBB dysfunction/permeability.	– High-fat diets in animal studies show an increase in inflammatory cytokines IL-6 and TNF-α in the reward circuit, and altered dopamine and opioid signaling, promoting addiction-like behaviors. – Human studies show that short-term exposure to high-fat/high-sugar increases inflammatory cytokines and enhances mesolimbic reward activity without weight gain, suggesting that UPFs have a direct effect on rewiring brain reward circuits – Neuroimaging in humans shows	– Support further research into FA as a valid addiction model	(34)
–	– Neuroimaging shows individuals with UPF addiction exhibit similar neural responses as addiction–greater anticipatory reward, reduced consummatory reward, enhanced functional connectivity across reward processing regions – Chronic UPF intake is associated with disrupted dopaminergic signaling and dysregulated hunger/satiety hormones	– Global prevalence of UPF addiction estimated at 14% of adults and 15% of youths (from two systematic reviews across 36 countries) – Individuals with obesity seem to be at higher risk for UPF addiction, with prevalence estimated at 28% of adults and 19% of youths with obesity	– Harm-reduction approach to overcoming UPF food addiction – Develop resources for clinicians, such as interviews for assessing and diagnosing UPF addiction	(132)
–	– High fructose corn syrup can cause gut dysbiosis, glucose tolerance, lipid neogenesis and oxidative stress -> dysregulation of key satiety hormones (leptin, ghrelin, insulin) and impaired signaling to arcuate nucleus of the hypothalamus. Repeated consumption can blunt satiety signals. – UPFs cause dependence similar to what is observed with addictive substances	–	–	(133)
–		FA mediated by salt, fat, sugar, caffeine Fructose can chronically inhibit leptin function, disinhibit ghrelin, fostering overconsumption –> sugar can activate the reward circuit, but chronic fructose exposure downregulates dopamine receptors, thus requiring more stimulus and more consumption, –> addiction		(136)
–	Refers to a paper on UPF having a high GI, which leads to triggering addictive processes	– Consumption of UPF is significantly associated ^**^ with food addiction	UPF increases FA risk, and minimally processed food reduces Considers if FA is related specifically to UPF	(135)
	– Processed foods facilitate addictive intake patterns similar to substance addiction corn syrup, thickeners, and glazing agents intensify flavors and optimize textural properties to be highly palatable	Increasing proportion of UPF related to FA, % increase in UPF and % reduction in minimally processed foods is significantly associated with reduced FA symptoms	Consider FA like substance addiction due to the high palatability of foods	(134)
**Eating disorders, food addiction**				
–	– UPF addiction involves reward-related neural dysfunction, impulsivity, and emotion dysregulation. – High-carb and added-fat UPFs trigger striatal dopamine responses similar to addictive substances; carb-fat combo has synergistic effect on reward. – Altered food matrix → faster consumption, greater bioavailability → upper intestine signals dopamine → more addictive. – Additives (artificial flavors, texturisers) enhance flavor and mouthfeel. – Low-socioeconomic areas → high UPF availability → greater reliance and higher UPF addiction.	– Pooled prevalence of food addiction across 281 studies using YFAS was 14% in adults and 12% in children, similar to the prevalence of other addictions such as alcohol and tobacco, suggesting that food addiction can be considered a real and valid addiction – Food addiction forms a significant percentage of eating disorders and obese individuals (32% in people with obesity having bariatric surgery and over 50% in those with binge eating disorder) – Among people with eating disorders or obesity, the presence of UPF addiction is linked to more severe clinical presentations and worse treatment outcomes	– Recommend recognizing UPF food addiction as an official diagnosis to promote research into clinical management, as a substantial proportion of people are being missed in current diagnostic frameworks, which only include eating disorders – Potential treatments include GLP-1 agonists and substance abuse drugs such as naltrexone and bupropion – Regulation needed to safeguard against industry practices that seek to maximize profit	(121)
–	– Reward system dysregulation: UPFs cause repeated dopamine release, leading to tolerance of reward systems over time, hence reinforcing binging behaviors – UPFs may amplify brain stress circuitry in such a way that continued intake becomes obligatory to prevent the development of negative emotional states via negative reinforcement – Excessive UPF consumption alters the brain circuits implicated in reward, decision making, control, habit formation, and emotions that are also implication in drug addiction -> supporting the theory of food addiction as an addictive disorder	– Loss of control binge eating (LCBE) is the hallmark of binge-type eating disorders (BED, bulimia nervosa, anorexia binge-purge type), within which UPFs are highly implicated–neurobiological studies show that individuals with LCBE show hyperactive reward systems, stress reactivity and cognitive impairment mirroring SUD – Significant overlap between the concept of food addiction, binge-type eating disorder and other substance addiction disorders	– Apply harm reduction and/or abstinence protocols for UPFs (UPFs) in treating binge-type eating disorders, as is commonly practiced in SUD. – Suggests changing the “all foods fit” approach to ED recovery to exclude or significantly reduce UPFs	(47)
–	–	UPF are highly palatable due to the taste enhancer ingredients		(130)
–	–	– Great overlap between SUDs and BN/BED, including reward dysfunction, emotion dysregulation, and impulsivity – Dominance of UPF in diet similar to addiction epidemic leading to increases in BED, obesity and cardiometabolic diseases	– Suggests that education and treatment alone are insufficient to overcome UPF addiction -> need policy initiatives such as increased taxation and limitations on product availability – Emphasis on protecting youth, e.g., through restricting marketing	(128)
–	–	Additives in UPF that improve flavor and texture can stimulate dopamine, similar to substance use –> evoke food craving, and trigger binge episodes	–	(131)
–	– Dopamine downregulation – Increases in glucose and insulin levels, which can contribute to overeating	UPF is associated with reward regions similar to drugs	–	(129)
–	Leptin and ghrelin levels were not significantly altered Involvement of the reward centers is proposed, visual stimuli of UPF and association with increased energy and increased intake UPF have faster transit times, meaning mechanisms to signal satiety and stomach stretching are slower, causing overeating	UPF is associated with increased eating rate and increased energy intake	Recommend against UPF consumption in food addiction, eating disorders	(126)
	– UPF alter satiety and allows for overconsumption	UPF consumption is significantly associated with ED and FA Dose-dependent response found	–	(123)
–	– UPF can be a form of “addiction transfer” and a coping mechanism	Statistically significant correlation of UPF vs. PTSD, FA, ED (individually) Interrelation between PTSD, FA and ED present UPF was a predictor of FA	Adverse childhood events can be a predictor of UPF intake, which can lead to future mental health problems	(91)
**Others**				
–	–	UPF associated with common mental disorders^**^, tertile 2 aOR 1.22 (1.11–1.33) and tertile 3 aOR 1.68 (1.51–1.87)	Potential bidirectional relationship between CMD and UPF	(185)
–	–	– Unhealthy eating patterns had a 9% higher prevalence of CMD, a significant association between unhealthy patterns vs. CMD^*^	–	(186)
–	Chronic low-grade inflammation Oxidative stress, processed diet may have minimal antioxidants BDNF is necessary for neuronal survival and plasticity	Soft drinks are associated with elevated CRP	–	(187)
–	–	No association between consumption of UPF and common mental disorders	–	(188)
	– Aspartame exposure changed glutamate–GABA signaling and gene expression in the amygdala, producing anxiety-like phenotypes—effects that persisted into subsequent generations descended from exposed males.	– Aspartame results in anxiety-like behavior in mice		(111)
		Maternal UPF consumption is associated with mental disorders		(189)

^*^Refers to statistical significance with *p* < 0.05. ^**^Refers to statistical significance with *p* < 0.01.

UPF were highly represented in the diets of patients with ED, and bulimic eating and binge-eating episodes were significantly associated with UPF consumption (119, 122). Binge episodes nearly exclusively contained UPF (47). Further studies have demonstrated that UPF overconsumption is associated with increased eating rates and higher energy intake (125, 126). The consumption of salty ultra-processed foods, including fried snacks, pizza, and fast foods, was associated with higher levels of loss of control eating and overeating (125). Similarly, sweet ultra-processed foods and sugar-sweetened beverages were also linked to loss of control eating and overeating. Sex-specific associations were observed, with salty food intake in girls and sweet food intake in both boys and girls associated with maladaptive compensatory behaviors, including laxative misuse and self-induced vomiting (125, 127).

#### 3.4.3 Mechanisms linking UPF consumption and eating disorders

A range of mechanisms has been proposed to explain the strong association between UPF intake and ED, involving various neurobiological, metabolic, and behavioral pathways (Table 2).

##### 3.4.3.1 Neurobiological pathways

The consumption of UPF may, in some cases, lead to the rewiring of the brain's reward systems, and these synaptic changes may replicate the types of changes that lead to reward and emotional dysfunction in substance use disorders (SUD) (128, 129). LCBE leads to the hyperexcitability of reward systems, increased stress reactivity, and cognitive impairment (47). UPF may also contribute to increased food intake as a result of increased stress activity (47). These changes may lead to a range of changes across dopamine and serotonin neurotransmitter systems across a wide range of brain networks (88, 124, 125, 129). UPF, which consists of carbohydrates and fats, have several important functions which lead to several synergistic actions which alter the brain's reward system (121). The dysregulation across the different neurotransmitter systems may contribute to the formation of habitual and inflexible unhealthy food choices, which have been implicated in the development of BED (24). Among individuals with BED, dysregulation across these neurotransmitter systems may result in a range of changes involving the orbitofrontal cortex and inferior frontal gyrus (124). Changes in the brain's reward pathways may also lead to new UPF cue-induced cravings (88) which can, in some cases, be triggered by a visual trigger (129).

##### 3.4.3.2 Additives and palatability

Additives in UPF, such as added salt, sugar and flavoring, increase the palatability of foods (121, 122, 124, 125, 127, 130). The additives may activate dopamine, which contributes to cravings and binge episodes (131). One of the purported mechanisms responsible for these changes is that the additives in UPF may interact with the striatum and hippocampus, which are known to assess the local calorie content, food taste and flavor (119, 120). The additives may disrupt the brain's ability to perceive the caloric content, taste, and flavor of food, leading to overconsumption (88, 122). Moreover, disruption in the striatal circuits has been found to promote the formation of habits and compulsive eating patterns (24). The additives contained in UPF may also cause neuroinflammation (124).

##### 3.4.3.3 Metabolic and hormonal pathways

UPF has also been linked to changes in insulin sensitivity and satiety, which may also occur in the case of ED (118, 123, 129). Changes in the food matrix may lead to improved absorption and release of dopamine (121). However, hormonal involvement has also been identified as contributing to changes across different neurotransmitter systems (118, 119). Furthermore, increased levels of ghrelin, fasting glucose, and insulin, as well as a reduction in the peptide tyrosine (appetite-suppressing), were found among individuals who had a diet high in UPF (118, 119). In addition, reduced levels of leptin were associated with higher rates of hedonic eating (88). However, Ulug et al. (126) investigated individuals who were on a diet with a high dietary content of UPF. They found no significant changes in the levels of leptin or ghrelin (126).

Some studies have shown that AN may be associated with reduced insulin sensitivity (119). Furthermore, there have been some studies which have shown that BN and BED were associated with increased levels of insulin resistance (119). In fact, artificial sweeteners were associated with higher levels of glucose intolerance (118). Insulin resistance and hyperinsulinemia have been found to promote hunger, contribute to cravings and lead to overeating (118, 129). Some adverse metabolic effects of UPF include changes in lipid composition, resulting in heightened levels of inflammation, endothelial dysfunction, and increased fat, which may, in turn, contribute to increased insulin levels (24).

Other mechanisms that have been implicated as being important to the development of adverse metabolic effects include the maternal consumption of UPF, which may lead to changes in the fetal BBB, among individuals with ED (120). Furthermore, UPF contain reduced levels of fiber content, which may facilitate increased rates of eating as well as an increased consumption of UPF, which in some cases leads to a significant reduction in the levels of satiety (122).

Hence, these recent studies have demonstrated that UPFs have an important and multifaceted role in the onset and maintenance of ED, particularly in terms of the development of BED and BN. Their impact extends beyond calorie load to involve neurobiological alterations, metabolic dysregulation, and behavioral reinforcement of maladaptive eating. Early exposure to UPFs may further predispose individuals to ED, highlighting the importance of dietary interventions in prevention. These findings emphasize that reducing UPF intake could serve as a modifiable risk factor for ED, complementing psychological and medical approaches to prevention and treatment. Moreover, a range of studies provides increasing evidence for integrating the assessment of specific dietary patterns and their frequency into clinical risk assessments, particularly among at-risk populations, to enable earlier and more targeted therapeutic interventions.

### 3.5 UPF and food addiction

#### 3.5.1 Background on known mechanisms of food addiction

Based on previous studies, it has been found that UPF addiction is present among approximately 14% of adults and 15% of youths globally, although UPF addiction has been found to have a higher prevalence among obese individuals (132). FA and ED, including BED, are typically considered as being separate diseases, but may, in some cases, co-occur (121, 128). Among individuals with ED and concurrent obesity, individuals may have higher rates of severe disease as well as more severe long-term outcomes (121). A range of proposed hypotheses suggests that food addiction and substance addiction may involve similar brain networks (47, 121, 128, 133, 134). Furthermore, UPF may be a form of “addiction transfer” and, in some cases, be a type of coping mechanism in view of its underlying association with post-traumatic stress disorder (PTSD) (91).

#### 3.5.2 Effect of UPF on the increased risk of food addiction

The consumption of UPF was significantly associated with FA (91, 123, 135), and a dose-dependent increase in FA risk (123) (Table 2). The proportion of UPF in the diet was also associated with an increased risk of FA (134). Furthermore, higher levels of UPF were associated with an increased eating rate and energy intake (126). Some of the identified food components associated with an increased risk of FA included salt, fat, sugar, and caffeine (136).

#### 3.5.3 Mechanisms linking UPF with food addiction

Some of the key mechanisms in which UPF may contribute to FA include its effects on the reward system (47, 121, 128, 129) (Table 2). There have been previous neuroimaging-based studies that have shown similar reward patterns and loss of control deficits among individuals with FA and substance use (132, 137). Consumption of UPF resulted in heightened levels of reward activity in the caudate and anterior cingulate regions (137). This may be mediated by the high palatability of UPF (137).

The carbohydrates associated with UPF may be addictive due to their stimulation of dopamine (121, 137). The refined carbohydrates result in a spike in blood glucose levels and a delay in dips in glucose levels. They also cause the release of insulin and may result in a hypoglycemic state, which can lead to a greater activation of the reward centers, leading to an increased urge for overconsumption of UPF (137).

UPF are high in saturated and trans fats, which stimulate a greater reward than foods containing natural fats (137). Furthermore, UPF enhance the palatability and inhibits the effects of high sugar intake as a consequence of reducing the glycaemic index and withdrawal response from UPF (121, 137). The high fat content results in elevated levels of dopamine across the reward circuits of the brain (138). The combination of carbohydrates and fats in UPF leads to a “supra-addictive reward response” (121, 137). Fats can affect and, in some cases, modify the reward pathway by increasing the levels of the inflammatory cytokines IL-6 and TNF-alpha (34). Saturated fats may also activate TLR4s, leading to the stimulation of the inflammatory pathways and cytokine release (34). These cytokines may cross the BBB, thereby inducing neuroinflammation across the reward pathway and altering synaptic plasticity, dendritic spine formation and myelination (34). The high-fat diets may, in turn, influence the integrity of the BBB, leading to elevated levels of serum albumin, which is an observation based on studies involving the analysis of an individual's cerebrospinal fluid (CSF) (34). A high-fat diet may, in turn, lead to the direct stimulation of inflammation or indirectly affect inflammation through the impact of a high-fat diet on the white adipose tissue (34).

The hypothesis that UPF may, in some cases, contribute to FA is supported by other findings that have demonstrated tolerance and withdrawal symptoms associated with the consumption of UPF. UPF addiction has been found to lead to neural responses that are similar to those that are seen in substance addiction, and these responses include a greater anticipatory reward, reduced consummatory reward, and enhanced functional connectivity in reward processing neurons (132). Continuous UPF consumption may also lead to a blunting of emotional states (47). The prolonged consumption of sugars such as fructose (136) and sucrose (47, 138) may also lead to a reduction in the dopamine response as a consequence of the downregulation of D2 receptors, thereby leading to tolerance, which is a finding that has been found across both human and animal studies (99, 135, 137). Furthermore, chronically high lipid levels consumed in the context of high-fat diets may reduce dopamine levels in the nucleus accumbens (138). Altered dopamine and opioid receptor signaling, which are like those seen in substance use and addictive disorders, have been observed in high-fat diet animal studies (34, 132). In animal-based studies, some withdrawal studies have included tremors, cravings, and increased stress that arise as a consequence of increased CRF levels (99). Reduced levels of dopamine in the nucleus accumbens were also reported (99), alongside altered dopamine signaling (88). Thus, UPF may lead to an alteration of dopamine and serotonin signaling similar to what is seen across SUD (129, 131).

Altered levels of satiety may contribute to UPF, leading to FA and overconsumption of UPF (123). In particular, corn syrup, thickeners, and glazing agents intensify flavors and palatability (121, 130, 131, 134). High fructose corn syrup may contribute to changes in gut dysbiosis, glucose tolerance, lipid neogenesis and oxidative stress (133). These changes may lead to a satiety hormone imbalance and signal impairments in the hypothalamus, which are involved in satiety (133). Nevertheless, there has been some dispute about the role of UPF signaling on leptin and ghrelin. Notably, Ulug (126) found that leptin and ghrelin levels were not significantly altered, but Lustig (136) has found that fructose inhibits leptin and stimulates ghrelin, resulting in increased hunger and overconsumption. Additionally, Wiss and LaFata (88) found that UPF was associated with reduced leptin levels, hedonic eating, and decreased satiety. Food cravings have also been found to be stimulated by UPF consumption (88, 126, 131). Reduced glucose and insulin levels from UPF have been linked to overeating in animal studies, although further studies in humans are needed (129).

Altered food matrices may, in some cases, lead to UPF being easier and faster to consume, with greater levels of bioavailability (121). That is, UPF is absorbed more quickly and able to alter levels of dopamine signaling, thereby leading to its addictive actions (121). The rapid consumption and transit of UPFs have been shown to impair dopamine-mediated satiety signaling, delaying the brain's recognition of fullness. This can result in gastric distension and contribute to the overconsumption of UPFs (126).

### 3.6 UPF and attention deficit hyperactivity disorder

#### 3.6.1 Background on known mechanisms of ADHD

The exact etiology and pathophysiology of ADHD remain incompletely understood. However, current evidence suggests that the disorder arises from a complex interplay of genetic predisposition, altered monoamine neurotransmission, and structural brain abnormalities, which together increase susceptibility. Genome-wide association studies (GWAS) have identified numerous genes associated with ADHD, including those involved in neurodevelopment (e.g., *FOXP2*), synaptic function (e.g., *GRM7, SORCS3*), and neuronal signaling pathways, particularly those regulating dopamine and serotonin receptor activity (139–141). Collectively, this polygenic component may account for approximately one-third of the heritability of ADHD (142). In addition to genetic vulnerability, dysfunction of dopamine signaling within the prefrontal cortex has been highlighted as a central mechanism underlying weak executive control and attentional deficits (143). Evidence suggests that an imbalance between tonic and phasic dopamine signaling, characterized by reduced baseline activity but exaggerated transient reward responses, leads to abnormally strong reward reinforcement effects and manifests clinically as impulsivity and distractibility (144). Structural brain differences further reinforce this picture. Neuroimaging studies show reduced volume in the frontal lobes, striatum, and interconnecting white matter in individuals with ADHD, which may be attributed to delayed cortical maturation (145, 146). Such alterations disrupt the integrity of fronto-striatal circuits, critical for behavioral regulation, thereby contributing to the hallmark symptoms of hyperactivity, impulsivity, and inattention (145, 146). Further research is needed to explore whether UPFs may interact with these pathophysiological pathways.

#### 3.6.2 Effect of UPF on the increased risk of ADHD

Current evidence based on a limited number of studies indicates that diets high in UPFs are consistently associated with increased ADHD symptoms and risk (Table 2). Early exposure, whether maternal consumption during pregnancy or intake during early childhood, appears particularly influential, correlating with higher scores of hyperactivity, inattention, and overall ADHD symptomatology (147–149). Significant food groups include sweetened beverages and sweets (149, 150). In contrast, children with higher overall diet quality, characterized by reduced UPF intake and greater consumption of minimally processed foods, show lower symptom burden and reduced likelihood of an ADHD diagnosis (147). UPF intake and ADHD risk also appear to follow a dose-dependent relationship, with greater frequency of consumption corresponding to higher symptom severity and increased diagnostic risk (150). While some associations are weak, with only a minor increase in risk, the overall trend suggests that repeated exposure to UPFs during fetal and childhood development may contribute to the development or exacerbation of ADHD (147, 149). Further studies are needed to consolidate these findings and explore effects in other populations, such as adults.

#### 3.6.3 Mechanisms of association between UPF intake and ADHD

Increasing evidence suggests that UPF consumption may contribute to ADHD risk and symptom expression through multiple biological pathways, including lipid metabolism, epigenetic programming, exposure to neurotoxins, and dopaminergic dysfunction (Table 2). One well-established factor in ADHD pathophysiology is the role of omega-3 fatty acids, which are crucial for maintaining neuronal membrane integrity and regulating neurotransmission. Individuals with ADHD consistently exhibit lower circulating levels of DHA, EPA, total n-3 PUFAs, and AA (148). Conversely, omega-3 supplementation has been shown to alleviate ADHD symptoms, suggesting a potential neuroprotective role in both the onset and progression of the disorder (148). Diets high in UPFs, typically deficient in omega-3 fatty acids, may further deprive the developing brain of these critical substrates. While evidence highlights omega-3 pathways as central to ADHD, further research is needed to clarify the contributions of other lipid pathways to disease mechanisms.

UPF intake during critical pre- or perinatal periods may also drive risk via epigenetic modifications. For example, high-fat/high-sugar maternal diets have been shown to alter methylation of the insulin-like growth factor gene, which plays an essential role in fetal growth and neurodevelopment, potentially predisposing to ADHD in childhood (147). Epigenome-wide association study (EWAS) in children similarly demonstrates that high-UPF diets are linked to DNA methylation changes in several genes relevant to neurodevelopmental disorders and behavioral regulation (36). Furthermore, contaminants within UPFs may contribute to pathogenesis. BPA, a plastic contaminant known to cross the placental barrier, has been implicated in behavioral disturbances, including hyperactivity (24). Similarly, heavy metal contaminants are also known to contribute to the development of ADHD. High intake of UPFs may cause dysfunction of the metallothionein genes and suppression of the paraoxonase-1 gene (PON1) (151). These neuroprotective genes are important for detoxifying heavy metals (151). UPFs are also characteristically high in refined sugars, which causes an immediate increase in glucose and adrenaline. This combination provides a short-term burst of energy, which can manifest as symptoms including excitement, impulsiveness and poor concentration consistent with ADHD (150). The longer-term effects of sugar can mimic dysfunction in the reward system. Chronic excessive sugar intake can lead to changes in mesolimbic dopamine signaling, via overstimulation of dopamine receptors and subsequent downregulation. This downregulation may result in reduced dopaminergic responses, which may in turn contribute to the development of addictive behaviors and are closely linked to the impaired cortical inhibition observed in ADHD (148).

### 3.7 UPF and autism spectrum disorder

#### 3.7.1 Background on known mechanisms of ASD

Autism spectrum disorder (ASD) is a complex neurodevelopmental condition characterized by persistent deficits in social communication as well as the presence of restricted, repetitive patterns of behavior and interests (152). Notably, ASD is the most common heritable neurodevelopmental disorder, with heritability estimates of approximately 80% (153). Inherited forms of ASD are often attributed to genetic mutations, in contrast to sporadic cases that may frequently arise as a consequence of microdeletions or duplications of chromosomal regions (154). These structural variations may overlap with the types of complex chromosomal changes observed across neurodevelopmental disorders, such as Angelman and Prader-Willi syndromes, as well as single-gene disorders, including neurofibromatosis (NF1 and NF2) and tuberous sclerosis (TSC1 and TSC2) (154). A broad range of genes have been implicated in the pathophysiology of ASD, including gene mutations that have been implicated in chromatin remodeling (e.g., CHD7), synaptic organization and function (e.g., SHANK family, neurexin), intracellular signaling pathways (e.g., G protein-coupled receptors, extracellular signal-regulated kinase (ERK) signaling), as well as several important genes that have been implicated across a range of different neurodevelopmental processes (154).

A number of previous studies that have specifically investigated the types of neurodevelopmental regression that have been seen in ASD have shown that these developmental changes may be associated with mitochondrial dysfunction, associated with impairment of oxidative phosphorylation and in some cases, this may lead to a disruption of the electron transport chain in the brain (155). These changes contribute to reduced energy production across several key brain networks, as well as increased levels of oxidative stress, which may, in turn, lead to significant disruptions across several key neurodevelopmental processes (156). In terms of other pathophysiological processes leading to changes in neural connectivity, several studies have demonstrated that alterations in the excitation-inhibition balance can result in a range of changes across various brain networks. In particular, abnormalities in glutamatergic and GABAergic neurotransmission across key brain regions such as the hippocampus, amygdala, and cerebellum may underlie several characteristic clinical features of ASD, including impairments in learning and memory, atypical visual perception (e.g., hypersensitivity to bright light), repetitive behaviors, and avoidance across social settings (157, 158).

Recent studies have implicated neuroinflammatory changes as being crucial in the development of ASD, with evidence of significant microglial activation and increased production of inflammatory cytokines and chemokines, including Interferon-γ (IFN-γ), IL-1β, IL-6, and TNF-α (159, 160). Furthermore, elevated levels of inflammatory cytokines have also been found in plasma, suggesting that peripheral inflammation may contribute to the development of ASD. These inflammatory markers have been linked to impairments in social interaction and communication, as well as aberrant behaviors seen in ASD (159). Although genetic factors have been implicated as contributing to a substantial proportion of ASD risk, a range of other environmental exposures have also recently been identified as potentially contributing to a heightened risk of developing ASD across the lifespan. Some of the important identified risk factors for ASD include advanced parental age, in utero exposure to drugs, toxins, alcohol, and tobacco smoke, as well as maternal disease and infection during pregnancy (161).

#### 3.7.2 Effect of UPF intake on increased risk of ASD

There continues to be a limited number of research studies that have specifically investigated the potential relationship between the UPF consumption and the subsequent risk of developing ASD, and further studies are needed (Table 2). There have been some studies that have identified a potential association between UPF consumption as well as the subsequent risk of developing ASD. For instance, high levels of UPF consumption have been found to be associated with an increased odds of developing ASD in adolescents, while experimental studies indicate that acrylamide, which is a compound frequently present in UPFs, may, in some cases, induce autism-like behaviors as a result of a number of important pathophysiological mechanisms that result in neuroinflammatory changes as well as oxidative stress (151, 160). These recent experimental findings are biologically plausible, particularly as both oxidative stress and neuroinflammation are increasingly recognized as important contributors to the pathophysiology of ASD (151, 156, 160).

However, not all studies have identified a particular association between the intake of UPF and the subsequent risk of developing ASD. In particular, Vecchione et al. (162) found that there was no significant link between consuming a UPF-rich Western dietary pattern and subsequently receiving an ASD diagnosis, suggesting that confounding factors such as genetic predisposition, overall dietary context, or methodological differences may explain inconsistencies across studies.

Overall, the findings from the current literature are conflicting and inconclusive, although they raise some important questions about whether certain diet-based factors, including diets with a high UPF exposure, may contribute to and influence neurodevelopmental outcomes across both healthy development and in the case of ASD. The possible mechanistic role of acrylamide and other UPF-derived compounds highlights the need for longitudinal human studies, as well as other animal-based studies, to elucidate the pathophysiological mechanisms involved and clarify whether the identified associations are causal. This avenue of translational research also has the potential to further clarify UPF's contribution to ASD risk, which could have significant public health implications, thereby informing dietary recommendations for children, adolescents, and pregnant women during critical neurodevelopmental windows.

#### 3.7.3 Mechanisms of association between UPF intake and ASD

There have been a range of recent studies that have shown that high dietary intake of UPF may contribute to an increased risk of developing ASD, which may be mediated through a range of different pathophysiological mechanisms, including lipid dysregulation, oxidative stress and neuroinflammation (Table 2). In a study that integrated findings from a lipidomic analysis, Hylén et al. (23) demonstrated that elevated inflammatory mediators were associated with reduced levels of endogenous antioxidants and, in particular, ether phospholipids. Based on these findings, it has been suggested that systemic low-grade inflammation may lead to significant oxidative stress through lipid dysregulation, which has been implicated in the pathophysiology of ASD (23). This was supported by findings from Ye et al. (160), in which administering an antioxidant (alpha-lipoic acid) was found to attenuate autism-like behavior in mice.

Furthermore, Ye et al. (160) investigated the effects of acrylamide (common in UPFs such as fried food) and demonstrated that high levels of damage to the native gut microbiome may result in a reduction in the production of SCFAs, while increasing levels of toxic LPS. In fact, previous studies have shown that serum LPS has various effects in the CNS, including the overactivation of microglia and an increased production of proinflammatory cytokines (116). These actions, resulting in neuroinflammation, are thought to lead to impaired synaptic plasticity as well as a range of other cognitive changes across key brain networks, which may contribute to the development of ASD. This may, in turn, manifest with a range of different traits, including learning and memory impairments (160). Further translational research is needed to investigate additional pathophysiological mechanisms and to build on the current findings, particularly in relation to the varied roles that genetic factors, early developmental factors, and environmental factors (e.g., exposure to toxins) may contribute to lipid dysregulation, as well as how these changes contribute to the development of ASD.

## 4 Discussion

This scoping review examined the association between the consumption of UPF and mental health–related disorders, with a specific focus on neurobiological mechanisms involving lipid metabolism. While previous reviews have linked UPF intake to adverse neuropsychiatric outcomes, these have largely relied on epidemiological associations or broad mechanistic frameworks. In contrast, this review uniquely synthesizes evidence directly interrogating lipid dysregulation as a central pathway linking UPF exposure to mental health outcomes. In comparison to prior narrative syntheses that propose putative mechanisms without systematically evaluating lipid pathways, the present review advances the field by integrating findings from experimental, preclinical, and translational studies to map how UPF consumption alters lipid synthesis, transport, and signaling within the central nervous system. These lipid disturbances are critical for neuronal membrane integrity, synaptic function, and neuroimmune regulation, providing a biologically plausible link between UPF intake and psychiatric vulnerability. Overall, the findings support a growing body of evidence associating high UPF consumption with multiple adverse mental health outcomes alongside consistent disruptions in lipid metabolic pathways. However, the specific causal mechanisms and their relevance across different psychiatric phenotypes remain incompletely defined. Among the proposed mechanisms, dysregulation of lipid metabolism appears to be a leading factor.

In terms of the relationship between the dietary intake of UPF and psychiatric disorders, the most robust and consistent evidence was observed for depression, with several key studies demonstrating a dose-dependent relationship between intake of UPF and subsequent development of depressive symptoms (46, 59–62). Higher levels of consumption of UPF during pregnancy were associated with an increased risk of depressive symptoms among offspring. In terms of specific food groups, it was found that the key food groups responsible for these changes included SSBs, fast foods, and fried foods (61, 65–70, 85). In the case of anxiety disorders, the evidence for an association between UFP and anxiety disorders was less consistent; only a few studies specifically investigated anxiety as an isolated outcome. Nevertheless, several studies reported an increased risk of developing an anxiety disorder when assessed in the absence of a comorbid mental health disorder, or in combination with a depressive disorder, or in the setting of another comorbid mental health disorder (67, 79, 94, 98, 114, 115).

There was also a strong association between the consumption of UPF and ED. ED, such as BN and BED, as well as FA, were associated with an increased eating rate, increased energy intake, as well as addictive reward responses, and these physiological changes were similar to the types of changes that were seen in the case of SUD (91, 122–126). There is emerging evidence to suggest that a high UPF intake may be associated with ADHD, which may adversely affect fetal and early childhood development, with sweetened beverages and sweets identified as key contributors (147–150). There have been other cross-sectional studies that have highlighted a potential association between the consumption of UPF and the risk of developing ASD. The association between UPF and the development of ASD may arise as a consequence of dietary contaminants in UPF, such as acrylamide, leading to changes in lipid regulation and metabolism (23, 151, 160).

### 4.1 Summary of key pathophysiological mechanisms

UPFs may lead to several pathophysiological changes across several important cellular and biochemical pathways, which have been implicated in the development of psychiatric disorders. In some cases, UPFs may adversely affect lipid profiles and compromise membrane integrity, thereby impacting BBB function. Reduced levels of SCFAs may lead to increased levels of BBB permeability, whilst TFAs have been found to increase phospholipid membrane rigidity, leading to altered neuronal signaling (24, 89). Oxidative stress resulting from the consumption of refined grains further reduces the fluidity of the lipid bilayer, thereby impairing neurotransmission (52). AGEs, such as acrylamide, may also contribute to elevated levels of endothelial dysfunction, oxidative stress, and inflammation (8, 97). Additionally, high omega-6 and low omega-3 PUFA intake has been found to promote systemic and neuroinflammation (117). Increased intake of SFAs can activate TLRs in the brain, which mimic bacterial LPS, triggering the activation of microglia and astrocytes, leading to the release of pro-inflammatory cytokines (35). In fact, UPFs may also disrupt various components of the gut-brain axis by increasing intestinal permeability, resulting in elevated circulating LPS, which contributes to heightened levels of peripheral and central inflammation (116). Some of the common pro-inflammatory markers implicated across mental health disorders include IL-1, IL-6, IL-17, IL-1β, and TNF-α (32, 93, 159).

In addition, UPF consumption has been implicated in metabolic disturbances such as hyperglycaemia and insulin resistance, which is a particular issue in the case of BED and BN. Furthermore, UPF commonly have higher glycaemic indexes, resulting in rapid spikes in blood glucose levels (84). These glycaemic fluctuations can influence gut microbiota composition by selectively promoting the growth of glucose-utilizing bacteria while suppressing beneficial fiber-fermenting species. This shift in microbial balance may alter the production of short-chain fatty acids and other metabolites, impacting intestinal barrier function, systemic inflammation, and host metabolic regulation.

These pathological changes drive hunger and cravings, reinforce overeating and contribute to inflammation and endothelial dysfunction (118). At the neurochemical level, UPFs alter serotonin and dopamine signaling, and these changes across these signaling pathways have been implicated in the development of depressive and anxiety disorders (52, 79, 91, 94, 98, 116). Furthermore, a glutamate/GABA imbalance has also been found to be associated with anxiety disorders, which provides further support that anxiety disorders may, in part, be caused by a disruption in the excitatory-inhibitory balance (111).

In summary, UPFs may have the potential to elicit addiction-like responses through overstimulation of dopaminergic reward pathways, which may lead to a downregulation of D2 receptors. These subsequent changes may lead to the development of abnormal tolerance patterns, which may be important in the development of SUD. These changes are evident in BN, BED, FA, and ADHD (88, 99, 124, 125, 129, 148). The synergistic combination of fats and refined carbohydrates and the destruction of the food matrix may, in some cases, accelerate the absorption of nutrients and amplify dopaminergic signaling (121).

### 4.2 Clinical implications

These recent experimental findings underscore the importance of incorporating diet-based factors, including dietary habits, into clinical practice to reduce the prevalence and adverse effects of UPF consumption. Hence, there is a recommendation for patient education regarding the types of harm posed by UPF consumption, with an emphasis on high-risk food subgroups, including fried foods, sugar-sweetened or artificially sweetened beverages, and processed meats (65, 67, 71, 74, 104, 125, 149, 150, 163). At a pragmatic level, clinicians should encourage practical substitutions, such as replacing sweetened juices with natural alternatives. Moreover, by considering contaminants from packaging, such as BPA, and additives including artificial colourings, like titanium dioxide, it is possible that appropriate measures can reduce the risk posed by these contaminants contained in UPF.

Educational programs should extend beyond the avoidance of harmful foods to include a comprehensive education program that promotes the health benefits of a balanced and healthy dietary intake. Diets which are rich in whole foods, such as the Mediterranean diet, and those with higher omega-3 fatty acid intake have recently been demonstrated as having a protective effect against mental health disorders, although further epidemiological studies are needed (8, 109, 148). Clinicians should continue to emphasize adherence to national dietary guidelines, which typically recommend whole-food-based eating patterns (164, 165). Raising awareness about the detrimental effects of UPFs may facilitate a shift toward preventive approaches in mental healthcare delivery.

Targeted education related to UPF consumption is important for vulnerable populations. Individuals with existing mental disorders should be informed about UPF as a risk factor for mental health conditions. Maternal nutrition during pregnancy is vital due to its influence on neurodevelopmental outcomes. Further, early-life education for children and adolescents is also important in view of the increased vulnerability of children and adolescents to mental health conditions. University students and young adults are another priority group because poor dietary habits are more common among individuals exposed to stressful environments (166).

Based on these findings, we propose that there is a need to integrate nutrition-based interventions with other non-pharmacological therapies as part of evidence-based care across the spectrum of mental health disorders. For mental health conditions that have a relatively strong association with UPF consumption, including FA and ED (namely BN and BED), multidisciplinary-based interventions involving psychiatrists and dietitians, as well as other health professionals, may lead to enhanced patient outcomes. In view of the socioeconomic and lifestyle factors associated with UPF consumption, greater consideration should be given to multidisciplinary-based interventions when managing patients across a broad range of clinical settings, particularly as limited access to whole foods and constraints on cooking ability may be significant barriers to patients implementing appropriate dietary changes.

### 4.3 Implications for policy making and public health

The current review emphasizes the impact of lifestyle choices on overall health outcomes. Not only has UPF been implicated as adversely affecting cardiovascular and metabolic health, but there is increasing recognition about UPF's impact across several domains of mental health (102). Public health sector policy can help the population make informed choices regarding their diet. These policies should be developed in collaboration with a range of health professionals, such as nutritionists and neuroscientists, to develop multidisciplinary health-based interventions. Such policies may involve a range of incentives to reduce UPF consumption, such as a tax on UPF or marketing restrictions. Policies aimed at implementing reforms in the marketing of UPF could include regulations related to the types of advertising and processing included in packaging. Such policies have been implemented in several countries, including Brazil, Mexico, Chile, and South Africa (167). This measure could include several different actions, such as revisions to the NOVA classification scheme or the use of current health star ratings.

In addition, there are several drivers of UPF consumption, which include a range of demographic-related factors, including obesity (168). In fact, previous studies have found that low socio-economic status and areas with low food security are at higher risk of UPF consumption (5, 169, 170). Thus, policies should ensure the affordability and accessibility of whole and minimally processed foods, such as through community-based food production.

Therefore, education about the potential adverse impacts of UFP and the beneficial effects of minimally processed foods, as well as steps to improve diets, may have several beneficial long-term effects across a diverse range of communities. This could be achieved through school programs and community initiatives, utilizing targeted advertising. These would include how to interpret processed foods rating systems and the dietary guidelines for minimally processed foods.

### 4.4 Future directions

Future research studies should prioritize longitudinal cohort designs to examine the directionality of associations between UPF consumption and mental health outcomes. Randomized controlled trials that substitute UPFs with minimally processed or whole-food dietary patterns, such as the Mediterranean diet, could be useful in providing stronger therapeutic evidence by assessing the extent to which dietary modification may reduce the severity and/or mitigate the types of psychiatric presentations. Therefore, examining the purported dose-response relationships between UPF consumption and mental health conditions will be important as part of new studies to investigate the potentially “safe” thresholds of UPF consumption that minimize risk.

To reduce the heterogeneity of further findings, future studies should adopt a standardized food classification framework, including the NOVA system, to ensure consistency in defining and quantifying UPF (2). Moreover, further work aimed at incorporating psychiatric diagnoses rather than self-reported questionnaires can strengthen the validity and applicability of mental health outcome measures. Furthermore, the impact of mental health disorders should be assessed individually as opposed to using the non-specific outcome of “common mental disorders.” At the same time, there is a need for new biochemical studies to investigate how UPFs affect key pathophysiological pathways involved in the development of mental disorders, particularly how lipid dysregulation and changes in specific lipid species contribute to heightened levels of neuroinflammation and altered neurotransmission. Integrative translational approaches, which combine a range of investigative methodologies including neuroimaging, metabolic profiling, and inflammatory markers, could lead to new avenues of study and offer new insights into the detrimental effects of UPFs, as well as the efficacy of potential therapeutic interventions. Finally, further studies aimed at developing evidence-based, individualized interventions, including omega-3 fatty acid supplementation, have the potential to inform the development of new targeted interventions that mitigate the detrimental impact of high-UPF diets.

### 4.5 Limitations

As part of this review, important correlations were identified between the UFP and a range of mental health conditions. However, no longitudinal studies were used. Most studies were cross-sectional, which reduced the ability to make causal inferences as well as generalize the findings to the broader community. More prospective cohort studies could strengthen the evidence. Assessment of the outcomes following the intervention, consumption of UPFs, has yielded inconsistent findings. The cross-sectional studies incorporate a range of self-reporting measures of dietary intake, including food-frequency questionnaires or interviews, which can be inaccurate and prone to recall bias (171). Furthermore, the amounts of food and their categorization were not standardized across the several studies included in the review. The classification of “processed foods” varied, and not all studies utilized the NOVA classification; some studies grouped foods into customized dietary patterns. In addition, outcome measurements were also variable, as some studies measured symptoms of a mental illness, whilst others required a diagnosis. The diagnostic criteria also differed across the different studies.

Based on the findings, there is a need for further work aimed at unraveling the mechanisms through which UFP may contribute to the development of mental health conditions. Further RCT-based studies are not currently feasible; therefore, most studies have been conducted using animals. However, findings from these studies may be challenging to generalize to clinical populations. Conducting longitudinal studies is also challenging, as they could be helpful in assessing temporal relationships. Further work aimed at developing longitudinal-based studies to assess whether there may be a bidirectional relationship between UPF and mental health disorders offers a new avenue for further translational research studies. At the same time, the clinical populations included in the studies typically consisted of a younger cohort, and hence, it may not be easy to generalize the findings to an older clinical cohort. Furthermore, there is currently a lack of clear studies that have been conducted across the neurodevelopmental period, from in utero to the young child, where the brain is susceptible to environmental impacts, and potentially to the impact of UPF. Therefore, further studies are necessary to address this significant gap in the clinical research literature.

## 5 Conclusion

The global rise in mental health disorders has occurred alongside increasing consumption of ultra-processed foods, highlighting the need to better understand dietary determinants of brain health. This scoping review synthesized evidence published between 2020 and 2025 examining associations between ultra-processed food intake, lipid metabolic disruption, and mental health outcomes. Across studies, higher consumption of ultra-processed foods was consistently associated with an increased risk of depression, anxiety, attention deficit hyperactivity disorder, eating disorders, and food addiction, with largely dose-dependent relationships observed.

These associations are supported by biologically plausible neurobiological mechanisms. Dysregulation of lipid metabolism emerged as a central pathway, with UPF consumption linked to altered fatty acid profiles, neuroinflammatory signaling, and impaired neurotransmitter systems, particularly those involving serotonin and dopamine. Such lipid-mediated disruptions are especially relevant to mood disorders, eating disorders, and food addiction. Additional contributions from food additives, packaging-related chemicals, and gut–brain axis perturbations are also likely, although mechanistic evidence remains limited.

Importantly, these findings have clear translational and therapeutic implications. Dietary interventions that reduce ultra-processed food intake and prioritize minimally processed nutrient-dense foods may represent a feasible, low-risk strategy to support mental health by restoring lipid balance and reducing neuroinflammatory burden. Targeting lipid metabolic pathways through nutritional modification or adjunctive therapies may complement existing pharmacological and psychosocial treatments, particularly for disorders characterized by metabolic and inflammatory dysregulation. Future research should prioritize longitudinal and mechanistic studies to clarify causality, identify sensitive developmental windows, and determine whether dietary modification can be leveraged as a preventive or adjunct therapeutic approach. Collectively, the evidence supports integrating dietary quality into mental health prevention, clinical management, and public health policy, underscoring the importance of limiting ultra-processed food consumption while promoting whole, nutrient-dense dietary patterns.

## Data Availability

The original contributions presented in the study are included in the article/Supplementary material, further inquiries can be directed to the corresponding authors.
